# Tracking changes in adaptation to suspension growth for MDCK cells: cell growth correlates with levels of metabolites, enzymes and proteins

**DOI:** 10.1007/s00253-021-11150-z

**Published:** 2021-02-13

**Authors:** Sabine Pech, Markus Rehberg, Robert Janke, Dirk Benndorf, Yvonne Genzel, Thilo Muth, Albert Sickmann, Erdmann Rapp, Udo Reichl

**Affiliations:** 1 Bioprocess Engineering, Otto von Guericke University Magdeburg, Magdeburg, Germany; 2 Bioprocess Engineering, Max Planck Institute for Dynamics of Complex Technical Systems, Magdeburg, Germany; 3 Section S.3 eScience, Federal Institute for Materials Research and Testing (BAM), Berlin, Germany; 4 Leibniz-Institut für Analytische Wissenschaften – ISAS – e.V., Dortmund, Germany; 5 Medizinische Fakultät, Medizinisches Proteom-Center (MPC), Ruhr-Universität Bochum, Bochum, Germany; 6 Department of Chemistry, College of Physical Sciences, University of Aberdeen, Aberdeen, UK; 7 glyxera GmbH, Magdeburg, Germany

**Keywords:** MDCK cell, Suspension growth, Metabolism, Enzyme activity, Proteome

## Abstract

**Abstract:**

Adaptations of animal cells to growth in suspension culture concern in particular viral vaccine production, where very specific aspects of virus-host cell interaction need to be taken into account to achieve high cell specific yields and overall process productivity. So far, the complexity of alterations on the metabolism, enzyme, and proteome level required for adaptation is only poorly understood. In this study, for the first time, we combined several complex analytical approaches with the aim to track cellular changes on different levels and to unravel interconnections and correlations. Therefore, a Madin-Darby canine kidney (MDCK) suspension cell line, adapted earlier to growth in suspension, was cultivated in a 1-L bioreactor. Cell concentrations and cell volumes, extracellular metabolite concentrations, and intracellular enzyme activities were determined. The experimental data set was used as the input for a segregated growth model that was already applied to describe the growth dynamics of the parental adherent cell line. In addition, the cellular proteome was analyzed by liquid chromatography coupled to tandem mass spectrometry using a label-free protein quantification method to unravel altered cellular processes for the suspension and the adherent cell line. Four regulatory mechanisms were identified as a response of the adaptation of adherent MDCK cells to growth in suspension. These regulatory mechanisms were linked to the proteins caveolin, cadherin-1, and pirin. Combining cell, metabolite, enzyme, and protein measurements with mathematical modeling generated a more holistic view on cellular processes involved in the adaptation of an adherent cell line to suspension growth.

**Key points:**

*• Less and more efficient glucose utilization for suspension cell growth*

*• Concerted alteration of metabolic enzyme activity and protein expression*

*• Protein candidates to interfere glycolytic activity in MDCK cells*

**Supplementary Information:**

The online version contains supplementary material available at 10.1007/s00253-021-11150-z.

## Introduction

Adaptation of mammalian cell lines to growth in suspension in a chemically defined medium has significant advantages for the design and optimization of manufacturing processes for biologicals. It requires the cells to reorganize their physiology away from cell-cell and cell-surface interactions towards suspension growth. Proteomics is a powerful tool to track changes in the protein composition and expression that has already been used to investigate on the link between modified environmental conditions and physiological changes (Doolan et al. 2010; Kumar et al. 2008; Luz-Hernández et al. 2008; Meleady et al. 2011; Pascoe et al. 2007). In combination with biotechnological tools, which are widely used to study the influence of the medium on cellular metabolism and growth rate (Cruz et al. 1999; Genzel et al. 2005; Sussman et al. 1980), the behavior of cellular systems can be studied in more detail. However, so far, it is barely understood how concerted changes on the proteome, metabolome, and growth enzyme level compose a system response, which enables suspension growth. This is partly because of the highly complex mechanism of cell line adaptation and partly because of the immense number of samples to be analyzed and evaluated.

The adaptation of Chinese hamster ovary cells to suspension growth in chemically defined medium is a story of success that enabled high yields in the production of recombinant proteins (Bandaranayake and Almo 2014; Kildegaard et al. 2013). But there is no suspension cell line available, which allows for the establishment of a similar platform approach for viral vaccine production. In particular, only few suspension cell lines qualify for manufacturing. Due to strict host cell specificity of many viruses, even adherent cultivation conditions are still a pivotal element in vaccine production. Madin-Darby *canine* kidney (MDCK) cells efficiently propagate various influenza virus strains (Genzel and Reichl 2009; Gregersen et al. 2011). For this cell line, different successful adaptations to suspension growth have been reported. So far, the human siat7e gene expressing MDCK cells (Chu et al. 2009) as well as suspension cells derived from adherent MDCK cells of the American and European collection of cell cultures (Huang et al. 2015; Lohr et al. 2010; van Wielink et al. 2011) are available for research purpose and some are even used for manufacturing of influenza vaccines (Donis et al. 2014; Doroshenko and Halperin 2009; Genzel and Reichl 2009; Gregersen et al. 2011; Manini et al. 2015; Onions et al. 2010; Perdue et al. 2011).

Adherent MDCK cell lines (MDCK_ADH_) have been extensively studied regarding growth characteristics (Bock et al. 2009; Genzel et al. 2006; Mohler et al. 2008), extracellular and intracellular metabolite dynamics (Rehberg et al. 2014a; Rehberg et al. 2014b) including enzyme activity measurements (Janke et al. 2010b). The MDCK.SUS2 cell line (MDCK_SUS2_) was adapted earlier to growth in suspension by our group (Lohr et al. 2010). Until recently, analyses were mainly limited to a descriptive level and few factors affecting changes were observed (Kluge et al. 2015; Lohr et al. 2010). For the first time, we now combine analyses of growth behavior, enzyme activity measurements, and proteomics with model-based approaches to study the adaptation of cell lines to new growth conditions. In the first part of our study, cell growth of the MDCK_SUS2_ cell line was analyzed using a segregated model for cell growth (Rehberg et al. 2013a) providing specific growth rates, uptake rates, and yield coefficients. Afterwards, potential metabolic flux rates are compared to measurements of key enzyme activities and cross-checked with relative abundance from the proteome analysis to resolve shifts in central carbon metabolism. Finally, proteomic data were used to further analyze biosynthesis as well as cellular signaling to identify hints for metabolic alterations caused by cell line adaptation to growth in suspension in a chemically defined medium. All these parts are then brought together to track changes on different cellular levels and to identify interconnections and correlations.

## Materials and methods

### Modeling suspension growth

The model of Rehberg et al. (2013a) for adherently growing MDCK cells was adapted to describe growth in suspension. As cells originate from an exponentially growing pre-culture, the initial distribution of cells spreads over various diameter classes *X*_1_, …, *X*_5_ such that the increase in the total cell number (*X*_tot_) already satisfies ${\dot{X}}_{\mathrm{tot}}={\mu}_{\mathrm{max}}{X}_{\mathrm{tot}}$ at initial times of cultivation (see Online Resource 1) with *μ*_max_ as the maximum specific cell growth rate. In other words, we exclude the lag phase that is typically found after inoculation. Furthermore, water evaporation was excluded (*k*_evap_ = 0) and parameters related to *X*_tot_ or the volume of all cells (*V*^*C*^) are scaled to the medium volume. In particular, this applies to units of the cell growth specific yield coefficient of glucose (*Y*_*X*/Glc_) and glutamine (*Y*_*X*/Gln_), the medium volume–specific glucose uptake rate for maintenance (*m*_Glc_), the medium volume–specific glutamine uptake rate for maintenance (*m*_Gln_), and the approximate cell volume for larger times (${V}_{\ast}^C$). Since the citric acid cycle can be supplied from glutamate (Glu), the uptake of Glu was implemented as first-order rate law with the following:

1 $$ \frac{\boldsymbol{d}\left[\mathbf{Glu}\right]}{\mathbf{dt}}={\boldsymbol{k}}_{\mathbf{GLT}}\left[\mathbf{Glu}\right]\left(\mathbf{1}-\boldsymbol{f}\right){\boldsymbol{V}}_{\boldsymbol{S}}^{\boldsymbol{C}} $$

where *k*_GLT_ is the cell number-specific activity of the glutamate transporter, ${V}_S^C$ is the cell specific volume, and *f* is a growth inhibition factor. Accordingly, the glutamate transport is activated with an increase in *f* as already described (Rehberg et al. 2013b). A more detailed description of the model is given in Rehberg et al. (2013a), the source code of the model is provided in the Supplementary Material and parameters are listed in the Online Resource 1.

For model fitting, estimation of the parameter confidence intervals, and visualization of the results MATLAB© (Version R2007b, The MathWorks, Inc.) was used (Rehberg et al. 2013a). Determination of parameters was performed simultaneously on individual batches and ranges are reported where appropriate. Models and data were handled with the Systems Biology Toolbox 2 developed by Schmidt and Jirstand; integration of the ordinary differential equations was performed with the CVODE from SUNDIALS by Cohen and Hindmarsh. The algorithm Scatter Search For Matlab (SSm, (Egea et al. 2007)) was used for stochastic global optimization of parameters and initial conditions. All simulations were carried out on a Linux-based system.

### Experimental methods

Media, solvents, and buffers labeled aqueous (aq) were prepared with filtered water from a water purification system (Milli-Q Advantage A10, Millipore, Schwalbach, Germany). For mass spectrometric analysis, an additional filter unit to the water purification system was added to remove trace organics (LCPAK0001). Chemicals for cell cultivation had synthesis grade, and chemicals for mass spectrometry (MS) had MS grade. Experiments were performed at 21 °C room temperature if not explicitly stated.

#### Cell line and cultivation conditions

From the MDCK_ADH_, obtained from ECACC (No. 84121903), the suspension cell line MDCK_SUS2_ was derived following the protocol described by Lohr et al. (Lohr et al. 2010). For cultivation of MDCK_ADH_, GMEM (Gibco Invitrogen, Germany, No. 22100-093) and for cultivation of MDCK_SUS2_, SMIF08 medium (protein- and peptide-free, chemically defined medium available from Gibco Invitrogen (Germany) by contact through K. Scharfenberg, Fachhochschule Emden/Leer, Germany) was used as described previously (Peschel et al. 2013). Cell concentrations and viability were determined with a ViCell XR (Beckman Coulter, Germany) after trypan blue staining (filtered 0.4% w/v trypan blue with 150 mM NaCl (aq), Carl-Roth, Germany). In case of microcarrier cultivations, a hemacytometer was used to determine cell concentrations (Bock et al. 2009). Cell viability was always around 90% during passaging.

Stirred tank bioreactors were inoculated with either pre-cultures of MDCK_ADH_ cells cultivated in roller bottles (Cellmaster 850 cm^2^ from Greiner Germany, caps tightly closed, 250 mL medium, 4 days growth, 0.66 rpm,), or with pre-cultures of MDCK_SUS2_ cells cultivated in shaker flasks (baffled Erlenmeyer flask from Corning Incorporated Germany, vent 0.2 μm cap, 100 mL medium, 3 days growth, 185 rpm) at 37 °C. The actual cultivations were carried out in the cellferm-pro® system (DasGip AG, Germany), or in the BIOSTAT® B plus system (Sartorius stedim biotech, Germany) with a starting cell concentration of 0.3 × 10^6^ cells/mL in 1 L medium. For cultivation of MDCK_ADH_ cells, microcarrier (Cytodex 1, GE, Sweden) were added in a concentration of 2 g/L equal to about 8000 carriers/mL. The bioreactor was equipped with a pitched-blade stirrer operated at 50 rpm in case of MDCK_ADH_ and 75 rpm in case of MDCK_SUS2_. Temperature was controlled at 37 °C. Oxygen partial pressure was set to 40% by pulsed aeration with air enriched with 7.5% CO_2_ and 20% O_2_. The pH value of the media was controlled at 7.3.

Cellular cultivations were independently repeated five times for MDCK_SUS2_ and three times for MDCK_ADH_ cells. The first two batches with MDCK_SUS2_ cells in the BIOSTAT® B plus system were used for enzyme activity measurements and a strict sampling scheme was performed to determine cell concentration, pH value, and metabolite concentrations of glucose (Glc), Glu, glutamine (Gln), lactate, and ammonia. Metabolites were measured as described (Genzel et al. 2004). For follow-up cultivations, MDCK_SUS2_ and MDCK_AHD_ cells were adapted to growth in media with lower concentrations of Glu (1.5 mmol/L) and Gln (4 mmol/L) over three consecutive batches each. These batches were performed using both cell lines alternating in the cellferm-pro® system with minimal sampling for proteomic comparison.

#### Enzyme activity assays

Enzyme activities were quantified with three technical replicates each as described by Janke et al. (Janke et al. 2010a; Janke et al. 2010b). In short, cells were harvested at a cell concentration of 1.3 × 10^6^ cells/mL. After washing with ice-cold phosphate-buffered saline (200 g, 5 min, 0 °C), samples were frozen in liquid nitrogen and stored at − 80 °C. For direct and indirect enzyme activity assays, samples were kept on ice. Enzyme extraction was performed by sonication (maximum power, 30 s, Sonopuls HD2200, Bandelin, Germany) in a 1-mL extraction buffer (Janke et al. 2010b). Afterwards, the extract was centrifuged at 16,000 *g* for 5 min to remove cell debris. The supernatant was used to measure the respective enzyme activities as previously described.

#### Sample preparation for proteomic analysis

Preparation of all samples was carried out on ice. At 70 h of cultivation and a cell concentration of 1 × 10^6^ cells/mL (± 0.1 ×10^6^ cells/mL, *n* = 3), cells in a 100-mL medium were harvested by either carrier sedimentation (MDCK_ADH_ cells) or by centrifugation at 300 *g*, 5 min and 4 °C (swing-out rotor, type 7591, Heraeus, Germany; MDCK_SUS2_ cells). Cells in the supernatant of microcarrier cultures were also harvested, treated like MDCK_SUS2_ cells, and finally pooled with the carrier fraction. All samples were washed three times with Tris-buffer 30 mM (aq) Tris (Sigma-Aldrich, Germany), 150 mM (aq) NaCl (Merck KGaA, Germany), 1 mM (aq) phenylmethylsulfonylfluoride (Sigma-Aldrich, Germany), pH 7.6). Afterwards, cells were lysed at a ratio of 2:1 (v/v, sample to buffer) with sodium dodecyl sulfate–based lysis buffer (Tris-buffer, 0.5% (w/v, aq) sodium dodecyl sulfate (AppliChem GmbH, Germany), 1 mM (aq) magnesium chloride hexahydrate (AppliChem GmbH, Germany), 5 mM (aq) ethylenediaminetetraacetic acid disodium salt dihydrate (Sigma-Aldrich, Germany), and protease inhibitor (Complete Ultra Tablets, Mini Easypack, Roche Applied Science, Germany), 50 mM dithiothreitol. Lysate was homogenized by pipetting through a syringe (0.7 × 16 mm, Sterican Heparin, Tuberkulin, B. Braun Medical AG, Germany) and frozen at − 80 °C.

Samples were thawed at room temperature and treated for 30 min with benzonase (benzonase, 25 U/μl, nuclease, purity > 99%, CN: 70664-3, Millipore, UK) at a ratio of 1:2000 (v/v, sample to enzyme). All samples were filtered (pore size: 100 μm, Partec GmbH, Germany) to remove carriers from the MDCK_ADH_ cell samples. An ultracentrifugation step (360.000×*g*, 30 min, 20 °C) was performed to remove insoluble particles. Protein concentrations were measured with a BCA assay kit (Pierce, Thermo Scientific, Germany).

#### Proteome analysis

For proteome analysis, sample preparation was performed using a spin filter (Nanosep 10K omega, Pall Life Sciences, USA) to remove persistent interfering substances from the media (i.e., pluronic, (Manza et al. 2005). Therefore, the protein solution was applied to a filter unit (10 kDa, Pall, Germany), and centrifuged at 13.000 *g* for 10 min. After two washing steps with a 200-μL urea solution (8 M urea in 0.1 M (aq) Tris/HCl (AppliChem, Germany), pH 8.5), 100 μL of 50 mmol/L iodoacetamide solution (in urea solution, Sigma-Aldrich, Germany) was added. The filter unit was shaken for 1 min at 600 rpm (Thermomixer Comfort, Eppendorf, Germany), and incubated for 20 min in the dark. After centrifugation, three washing steps with a 100-μL urea solution and three washing steps with 100 μL 50 mM (aq) ammonium bicarbonate buffer (Fluka Analytical, Germany) were performed. Trypsin solution was added to the filter unit in a volume of 50 μL (50:1 (w/w, sample: trypsin), in ammonium bicarbonate buffer, with 5% (v/v) acetonitrile (ACN, Fluka Analytical, Germany) and 1 mM CaCl_2_ (AppliChem, Germany)). After shaking for 1 min at 600 rpm, the digestion was incubated for 7 h at 37 °C. The filter unit was placed onto another reaction tube. The filtrate was gained after centrifugation, and the filter unit was washed with a 50-μL digestion buffer (without trypsin) and 70 μL of water.

The filtrate was vacuum-dried (SPD 121P centrifuge, coupled to refrigerated Vapor Trap, Thermo Scientific, Germany) and re-suspended in 50 μL trifluoroacetic acid (TFA) solution for liquid chromatography (LC) separation (0.1% (v/v) TFA (Fluka Analytical, Germany), 2% (v/v) ACN). Digestion quality was tested with a 1-μL sample using monolithic high-performance LC (HPLC) separation (Ultimate 3000 nano LC system, Thermo Scientific, Germany) and UV detection at 214 nm as described (Burkhart et al. 2012). Sample solution was aliquoted in 2-μL volumes and vacuum-dried.

Dried samples were re-suspended in 0.1% (aq) TFA and equal peptide content was verified by the absorbance of the peptide bond at 205 nm with a NanoDrop2000 (Thermo Scientific, Germany). For nano LC−MS/MS analysis, a Q-Exactive mass spectrometer (Thermo Scientific, Germany) was online coupled to a nano RSLC HPLC system (Thermo Scientific, Germany). A total of 1.5-μg peptides (referred to the starting material of 50 μg) in 15 μl 0.1% (aq) TFA was injected. Samples were loaded onto a trap column (C18, 100 μm × 2 cm PepMap RSLC, Thermo Scientific, Germany) at a flow rate of 20 μL/min with 0.1% (v/v) TFA solvent. Subsequently, peptides were separated on a 50-cm main column (C18, 75 μm × 50 cm PepMap RSLC, Thermo Scientific) using a binary gradient consisting of mobile phase A (0.1% (v/v, aq) formic acid) and mobile phase B (0.1% formic acid, 84% ACN) at a flow rate of 250 nL/min and 60 °C. Gradients increased linearly from 3 to 42% B in 90 min. The Q-Exactive was operated in data-dependent acquisition mode acquiring full MS Scan in a mass range of 300–1500 m/z (*R* = 70,000) followed by MS/MS of the 15 most abundant ions (*R* = 17,500). Charge states +2 to +5 were selected for high energy collision dissociation fragmentation with a dynamic exclusion of 12 s and a collision energy of 27%. Target values and fill times were set to 3 × 10^6^ and 120 ms for MS and 5 × 10^4^ and 250 ms for MS/MS.

#### Proteome data evaluation

Label-free quantification was performed using the software Progenesis LC-MS 3.0 (Nonlinear Dynamics, Germany). LC-MS runs were aligned to an automatically selected reference run. After peak picking, a list of the features was exported and analyzed using SearchGUI (Vaudel et al. 2011). Therefore, the list was divided 15 times and analyzed using the OMSSA and X!tandem search algorithm through the Uniprot/Trembel database (2013/07) of *Canis familiaris* entries. The peak list from raw spectra, the list from the search engine MASCOT and the used database were uploaded at MassIVE (ftp://massive.ucsd.edu/MSV000086277/). Established search parameters: tryptic digest with a maximum of one missed cleavage; fixed modification—carbamidomethylation of cysteine, variable modification—oxidation of methionine; peptide charge 2+ to 4+; monoisotopic peptide masses; peptide tolerance of 0.3 Da; MS/MS tolerance of 0.5 Da. Data were imported into Peptide-Shaker (Version 0.22.5) and corrected with a false discovery rate of 0.05 (Barsnes et al. 2011; Vaudel et al. 2011). Afterwards, result files were imported to Progenesis and protein lists with calculated protein quantities were exported to Excel. For statistical analysis of the data, a Student’s *t*-test with a *p*-value < 0.05 was performed with R (R Core Team 2008).

For the evaluation of the proteome of MDCK_ADH_ and MDCK_SUS2_ cells, only proteins with at least 1.4-fold induction (summed up peptide peak intensities) of at least three unique peptides (software Progenesis LC-MS) were considered. Proteins were grouped according to the biological processes of the human homolog protein (translated by Uniprot BLAST (Consortium 2014) and bioDBnet (Mudunuri et al. 2009)) described in Uniprot (Consortium 2014).

## Results

### Cell growth

**Fig. 1:**
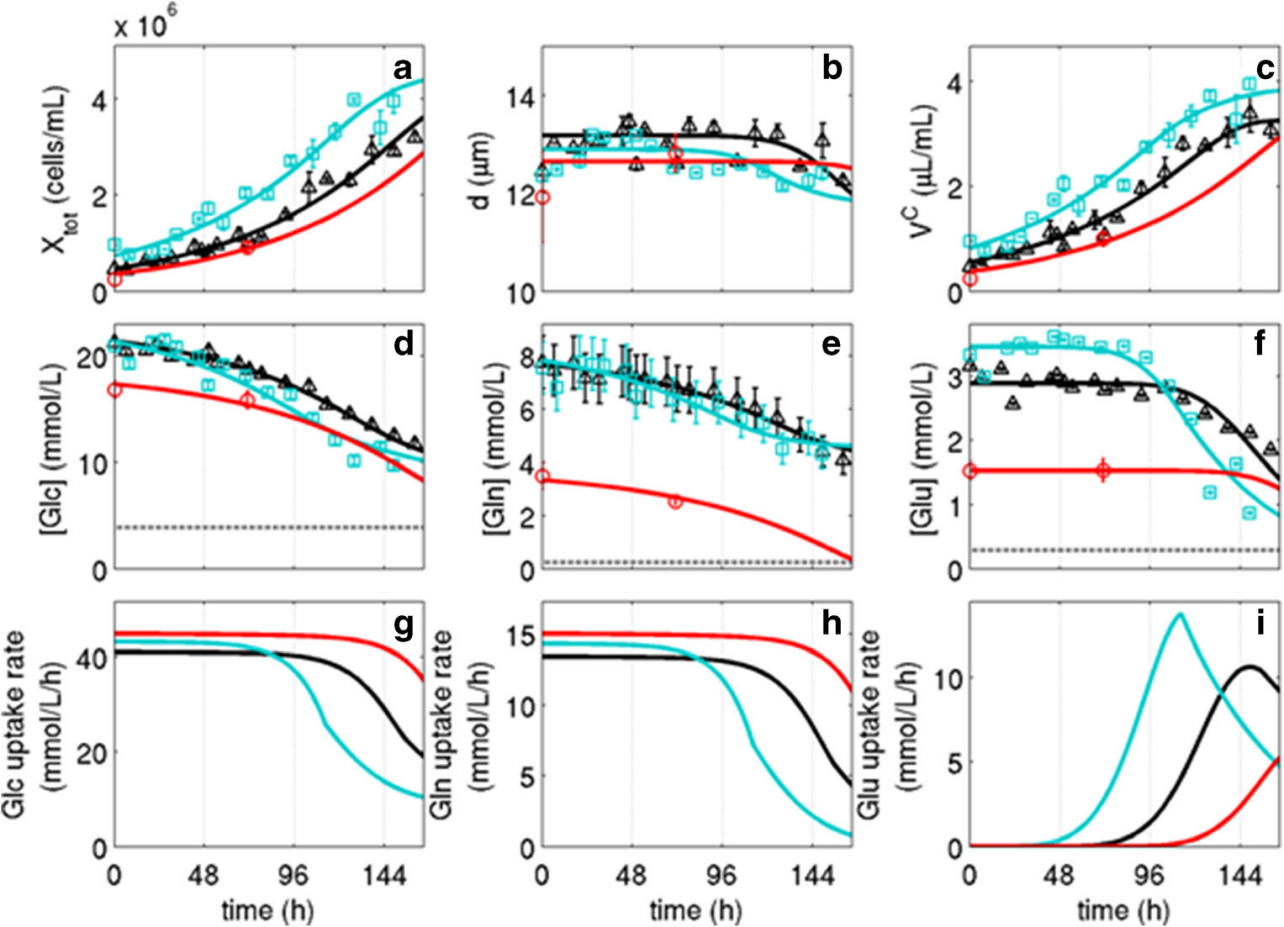
Growth of MDCK_SUS2_ cells with SMIF8 medium in 1-L-stirred tank bioreactor (5 batch runs). **a** Cell concentration *X*_tot_. **b** Mean cell diameter *d*. **C** Volume concentration of all cells calculated from the mean cell diameter and the cell number *V*^*c*^. Concentrations of glucose (**d**), glutamine (**e**), glutamate (**f**), and corresponding simulated cell volume–specific uptake rates for glucose (**g**) for glutamine (**h**) and glutamate (**i**). Data of the two independent experiments for enzyme activity measurements indicated as triangle (Δ) and rectangle (□). Data of the three independent experiments for proteomic analysis with minimal sampling were combined and average is indicated as circle (○). For all data shown, error bars represent mean and standard deviation of three technical replicates (in case of *V*^*C*^ error bars were calculated according to the error propagation law). Lines represent the corresponding model simulation (parameters of Table 1 and Online Resource 1). Gray-dotted line in D, E, and F indicates the limit of quantitation of the respective metabolite analysis

For the analysis of experimental results of MDCK_SUS2_ cell growth in stirred tank bioreactors, a model for MDCK_ADH_ cells described by Rehberg et al. (2013a) was adapted to characterize suspension growth. The model used cell growth-specific parameters (Table 1) and experiment-specific parameters (Online Resource 1). It provided growth rates, metabolic uptake rates, as well as yield coefficients and allowed an insight into the dynamics of MDCK_SUS2_ cell cultivation and a comparison with MDCK_ADH_ cell growth (Table 1). In general, the segregated model was in good agreement with the measured data points for cell number, Glc, Gln, and Glu concentrations (Fig. 1).

**Table 1:** Simultaneously estimated parameters of the cell growth models for MDCK_ADH_ cells (determined by Rehberg et al. (2013a)) in comparison to MDCK_SUS2_ cells (on the basis of five different cultivation runs) grown in a stirred tank bioreactor

Parameter		MDCK_ADH_	MDCK_SUS2_	Unit
*μ*_max_	Maximum growth rate	0.036 − 0.051	0.013 − 0.014	h^−1^
*Y*_*X*/Glc_	Cell growth–specific yield coefficient for glucose	(3.18 − 3.96) × 10^−6^	(0.72 − 0.78) × 10^−6^	mmol cell^−1^
*Y*_*X*/Gln_	Cell growth–specific yield coefficient for glutamine	(0.25 − 0.28) × 10^−6^	(0.24 − 0.37) × 10^−6^	mmol cell^−1^
*m*_*Glc*_	Medium volume–specific glucose uptake rate for maintenance	(0.92 − 1.19) × 10^−2^	0.2 × 10^−2 a^	mmol μL^−1^ h^−1^
*m*_Gln_	Medium volume–specific glutamine uptake rate for maintenance	≈0	Same as adherent	mmol μL^−1^ h^−1^
*Y*_LAC/GLC_	Glucose specific yield of lactate	2.034 − 2.283	0.86 – 0.99	-

^a^Not fitted. Projected from the cell volume–specific uptake rate of Glc

Cell concentration increased over the time from about 0.3 × 10^6^ cells/mL to a maximum of 4 × 10^6^ cells/mL (Fig. 1a). Maximum cell specific growth rates of 0.013–0.014 h^−1^ were observed and represent a reduction to 25–30% compared to MDCK_ADH_ (Table 1). Minor differences between the cultivations in initial cell concentrations increased over the time course of cultivations (Fig. 1a). Samples for enzyme activity measurements were taken at a concentration of 1.3 × 10^6^ cells/mL at 40 h and 58 h. The proteomic samples were taken between 60 and 70 h at a cell concentration of about 1 × 10^6^ cells/mL.

As the cell diameter stayed nearly constant during cultivations (Fig. 1b), the shape of the curves of cell number and cell volume were similar (Fig. 1a and 1c). Only towards the end of cultivations, the cell diameter slightly decreased, which is also described by the model. Initial changes in the cell diameter were small and probably attributed to the transfer of cells from one cultivation vessel to another, which resulted in a minor difference between data and model prediction (Fig. 1b).

Following the time course of cell volume, a transition from exponential growth to a growth inhibition phase is likely due the plateau forming at the end of cultivations (Fig. 1c). This growth inhibition might be related to the rather high cell densities or substrate limitations. A clear separation of growth phases usually seen for MDCK_ADH_ cells (Rehberg et al. 2013a) was not observed in these data.

### Metabolite pools and uptake rates

Over the time course of cultivations, MDCK_SUS2_ cells took up about half of the Glc (Fig. 1d). The decrease was relatively constant and ceasing towards the end of the cultivations. Regarding the Glc uptake rate, a distinct change was visible after three and four days (Fig. 1g). The calculated Glc uptake rate of 40 mmol/L/h decreased to 10 mmol/L/h. Compared to MDCK_ADH_ cell growth (Rehberg et al. 2013a), the Glc update rate and, at the same time, the cell specific yield coefficient for Glc was reduced to 20%, which implies less and more efficient glucose utilization for cell growth (Table 1). Accordingly, the specific yield of lactate from glucose was lower compared to MDCK_ADH_ with only 0.86–0.99 (Table 1), which represents a reduction to 40%. Average specific air flows of 0.11 day^-1^ and 0.20 day^−1^ were determined for MDCK_SUS2_ and MDCK_ADH_ cells, respectively (see Online Resource 2). This corresponds to a ratio of 0.36 and 0.24 for the maximum specific air flow rate to the maximum specific growth rate (MDCK_SUS2_ 0.11/0.31 day^−1^/day^−1^; MDCK_ADH_ 0.20/0.86 day^−1^/day^−1^). Accordingly, with all the caveats of air flow measurements, MDCK_SUS2_ cells seem to have a higher oxygen demand (50 %) during the exponential growth phase. MDCK cells convert the substrate Glc to lactate by aerobic glycolysis for the generation of cellular energy. A decreased activity of aerobic glycolysis for MDCK_SUS2_ cells, indicated by the generally lower Glc uptake rate and lower glucose specific yield of lactate compared to MDCK_ADH_, may indicate a higher energy supply by the citric acid cycle.

The substrate Gln decreased over the cultivation time, reflecting the demand by the cells, which is high during cell growth and low during cell maintenance phase (Fig. 1e). The decrease was constant with an anticipated flattening at the end of cultivations. Regarding the Gln uptake rate, a distinct change was visible after 3 and 4 days (Fig. 1h), similar to the Glc profiles. The Gln uptake rate of 15 mmol/L/h decreased to 1 mmol/L/h, which is slightly higher than for MDCK_ADH_ (12 mmol/L/h) with a somewhat higher cell specific yield coefficient for Gln (Table 1). Sources for citric acid cycle intermediates are intracellular Gln and Glu, while their conversion by glutaminolysis to *α*-ketoglutarate yields intracellular ammonium, which can be used for O-linked N-acetylglucosaminylation (Ryll et al. 1994) or can be directly transferred to other amino acids. The remains of ammonium was released as ammonia by the cells and yielded an increase in extracellular concentrations, which was similar for both cell lines (data not shown).

Glc and Gln uptake rates dropped after 3 and 4 days together with a decrease in Glu concentration (Fig. 1f). For MDCK_ADH_ cells, it was shown that the Glu transport was activated when the cells were growth inhibited (Rehberg et al. 2013a). The same correlation can be assumed for MDCK_SUS2_ cells as the Glu uptake rate showed a peak like behavior before the cell volume transitions from exponential growth to a growth inhibition phase (Fig. 1c and 1i). This phase was named as intermediate phase by Rehberg et al. (2013a), where the cells switch into maintenance metabolism. The maintenance substrate uptake rates were defined as the basal consumption that is required to sustain the actual biomass and were also simulated with the model (data not shown). The maintenance rate of MDCK_SUS2_ cells for Glc dropped to 20% compared to MDCK_ADH_ cells (Table 1). The maintenance rate for Gln was negligible, which indicates that glutamine was not required for cellular maintenance as also observed for MDCK_ADH_ cells (Table 1).

Differences between the three simulated cell cultivations are noticeable, especially for the mean of the three independent experiments performed for proteomic analysis (Fig. 1, red line). For the latter, concentrations of Glu and Gln were adapted to the cellular demand and halved in concentration compared to the two independent cultivations performed for enzyme activity measurements with a strict and comprehensive sampling scheme. To explain the drop of Glc and Gln uptake rates as well as the activation of Glu uptake (Fig. 1f), the mathematical model had to predict different timings of growth inhibition for the cultivations. However, the segregated model was in good agreement with all measured data points in a simultaneous use of identical growth parameters (Table 1), which highlights robust growth dynamics and a similar metabolic regimen.

### Altered maximum enzyme activities for MDCK_SUS2_ cells

The maximum catalytic activities of 27 enzymes from central carbon metabolism for MDCK_SUS2_ cells were measured during substrate saturation using an assay platform developed by Janke et al. ((Janke et al. 2010b), Table 2). The enzyme activities were measured in relation to the applied protein amount of cell extracts. At the sampling point with a cell concentration of 1.3 ×10^6^ cells/mL, the determined protein amount per 10^6^ cells was 0.22 ± 0.04 mg protein for MDCK_SUS2_ cells and 0.18 ± 0.01 mg protein for MDCK_ADH_ cells.

**Table 2:** Maximum activity and peptide peak intensity for metabolic enzymes for MDCK_SUS2_ and MDCK_ADH_ cells during the exponential growth phase in 1-L bioreactor cultivations

Enzyme	Enzyme activity in (nmol/min/mg protein)			Peptide intensity		
	MDCK_SUS2_	MDCK_ADH_^b^	Ratio MDCK_SUS2_/ MDCK_ADH_^c^	Peptides ^d^	Ratio MDCK_SUS2_/ MDCK_ADH_^e^	*p*-value ^f^
Glycolysis						
Hexokinase	**21.7 ± 2**	**83.8 ± 8.3**	**0.26**	**10 (4)**	**0.60**	***
Phosphoglucose isomerase	**374.5 ± 25.2**	**911.9 ± 42.9**	**0.41**	**22 (18)**	**0.83**	**
Phosphofructokinase	**23.1 ± 3**	**73.0 ± 2.7**	**0.31**	**11 (7)**	**0.63**	***
Triose-phosphate isomerase	**1022.7 ± 78.7**	**26,092.0 ± 3018.1**	**0.04**	**13 (12)**	**0.70**	***
Fructose-1,6-bisphosphate aldolase	**49.8 ± 2.8**	**145.4 ± 25.6**	**0.34**	**28 (17)**	**0.55**	***
Glyceraldehyde-3-phosphate dehydrogenase	**532 ± 32.9**	**1846.5 ± 272.0**	**0.29**	**31 (16)**	**0.67**	***
Phosphoglycerate kinase				13 (12)	0.45	***
Pyruvate kinase	**1330.3 ± 119.2**	**3479.9 ± 110.1**	**0.38**	**35 (30)**	**0.60**	***
Lactate dehydrogenase	**1308.3 ± 135.6**	**3596.5 ± 150.5**	**0.36**	**18 (17)**	**0.58**	***
Pentose phosphate pathway						
Glucose-6-phosphate dehydrogenase	**148.4 ± 9.7**	**337.6 ± 22.6**	**0.44**	**16 (16)**	**0.78**	***
6-Phosphogluconate dehydrogenase	**79 ± 3.5**	**183.7 ± 18.2**	**0.43**	**15 (13)**	**0.83**	**
Transketolase	34.7 ± 1.5	35.1 ± 4.4	0.99	20 (17)	1.26	**
Transaldolase	51.5 ± 2.6	76.9 ± 6.1	0.67	5 (3)	(1.16)	
Anaplerotic reactions						
Pyruvate dehydrogenase	0.2 ± <0.01	1.4 ± 0.2	0.12	8 (5)	1.46	***
Pyruvate carboxylase	1.3 ± 0.08	4.0 ± 0.3	0.32	6 (4)	1.63	***
Malic enzyme	**7.8 ± 0.6**	**44.1 ± 4.2**	**0.18**	**9 (7)**	**0.24**	***
Phosphoenolpyruvate carboxykinase	59 ± 8.5	191.8 ± 20.8	0.31	7 (6)	(1.01)	
Citric acid cycle						
Citrate synthase	42.8 ± 3	180.9 ± 7.7	0.24	ND		
Aconitase				20 (14)	1.47	***
NADH-linked isocitrate dehydrogenase	0.4 ± 0.1	0.5 ± 0.0	0.85	ND		
NADPH-linked isocitrate dehydrogenase	**191.1 ± 6.4**	**152.4 ± 13.2**	**1.25**	**6 (6)**	**1.76**	***
Succinate dehydrgenase				12 (9)	1.4	*
Fumarase	277.4 ± 22.4	419.5 ± 51.4	0.66	7 (5)	1.47	**
Malate dehydrogenase	2548 ± 222.5	4264.5 ± 294.1	0.60	12 (8)	(1.37)	
Glutaminolysis						
Glutaminase	**12.2 ± 0.6**	**33.6 ± 3.1**	**0.36**	**5 (5)**	**0.51**	***
Glutamate dehydrogenase	54.5 ± 3.7	22.4 ± 2.1	2.44	16 (13)	(1.09)	
Alanine transaminase	8.3 ± 0.9	28.1 ± 3.0	0.30	ND		
Aspartate transaminase	352.5 ± 27.4	683.6 ± 55.9	0.52	ND		
Miscellaneous						
Acetate-CoA ligase	**7.5 ± 0.6**	**5.2 ± 0.2**	**1.44**	**3 (3)**	**1.54**	**
Citrate lyase	**14.6 ± 1.3**	**24.6 ± 1.4**	**0.59**	**30 (24)**	**0.71**	**

^a^Calculated mean values for enzyme activity based on two independent cultivations (between 40 and 58 h) and protein expression based on three independent cultivations (between 60 and 70 h) with corresponding standard deviation (**±**). **Bold**, concerted alterations for both cell lines are highlighted (15 enzymes)

^b^Maximum enzyme activity of exponentially growing MDCK_ADH_ cells were taken from the publication of Janke et al. (2011) (Janke et al. 2011)

^c^Ratio for enzyme activity comparing MDCK_SUS2_ cells with MDCK_ADH_ cells

^d^Shown are peptide amounts detected with mass spectrometry and unique peptides used for protein quantification (in brakets) using the software Progenesis LC-MS

^e^Ratio for peptide intensity values (summed up for peptides for quantification using the software Progenesis LC-MS) comparing MDCK_SUS2_ cells with MDCK_ADH_ cells. Results in brakets were statistically not significant

^f^Statistical significance of the ratio for peptide intensity (*t*-test, *p*-value (*) < 0.01, (**) < 0.001, (***) < 0.0001)

Activities of the different enzymes covered four orders of magnitude (0.2 nmol/min/mg protein (pyruvate dehydrogenase) to 2548 nmol/min/mg protein (malate dehydrogenase)) for MDCK_SUS2_ cells. High activities (> 1000 nmol/min/mg protein) were found for malate dehydrogenase, pyruvate kinase, lactate dehydrogenase, and triose-phosphate isomerase, while the other enzyme activities were in the range of 1–400 nmol/min/mg protein. The lowest enzyme activities were found for pyruvate dehydrogenase (PDH), pyruvate carboxylase and NADH-linked isocitrate dehydrogenase (< 1.5 nmol/min/10^6^ cells), indicating possible rate-limiting steps in the metabolic pathway.

For a comparison of the MDCK_SUS2_ cell line with its parental MDCK_ADH_ cell line, the maximum enzyme activities measured by Janke et al. were listed in Table 2 (Janke et al. 2011), and the ratio MDCK_SUS2_:MDCK_ADH_ was calculated. A decreased maximum enzyme activity for MDCK_SUS2_ cells (ratio < 0.7) was determined for 22 enzymes, which included all analyzed enzymes for glycolysis and anaplerotic reactions, most analyzed enzymes of the pentose phosphate pathway, citric acid cycle, and glutaminolysis (two enzyme activities were equal). For three enzymes, glutamate dehydrogenase, acetate-CoA ligase, and NADP-linked isocitrate dehydrogenase increased maximum enzyme activities were found for MDCK_SUS2_ cells compared to MDCK_ADH_ cells (ratio > 1).

### Protein expression changes of metabolic enzymes

Comparative proteomic analysis between MDCK_SUS2_ and MDCK_ADH_ cells was focused to the metabolic enzymes analyzed before. Overall, 23 of the 27 enzymes were detected by the proteomic approach. Interestingly, for 65% of the enzymes, we observed significant changes in intracellular protein levels (Table 2, 15 enzymes, *p*-value < 0.05). For example, the intracellular protein level of two enzymes (glucose-6-phosphate dehydrogenase, 6-phosphogluconate dehydrogenase) of the pentose phosphate pathway was reduced in MDCK_SUS2_ cells compared to MDCK_ADH_ cells (Table 2), which might explain the differences in maximum enzyme activity. Also, differences in intracellular protein levels for acetate_CoA ligase (increase), citrate lyase (decrease), and glutaminase (decrease) correlated with the corresponding enzyme activity ratios (Table 2). On the other hand, for transketolase, a slightly higher intracellular level of protein was found (1.26) despite a similar enzyme activity ratio (0.99).

In contrast, changes in enzyme activities of anaplerotic reactions and of citric acid cycle seemed not to be related to intracellular protein levels (Table 2). In these pathways, only malic enzyme and NADP-linked isocitrate dehydrogenase show a concerted alteration of protein quantity and enzyme activity.

### Differences in intracellular protein expression levels

**Fig. 2:**
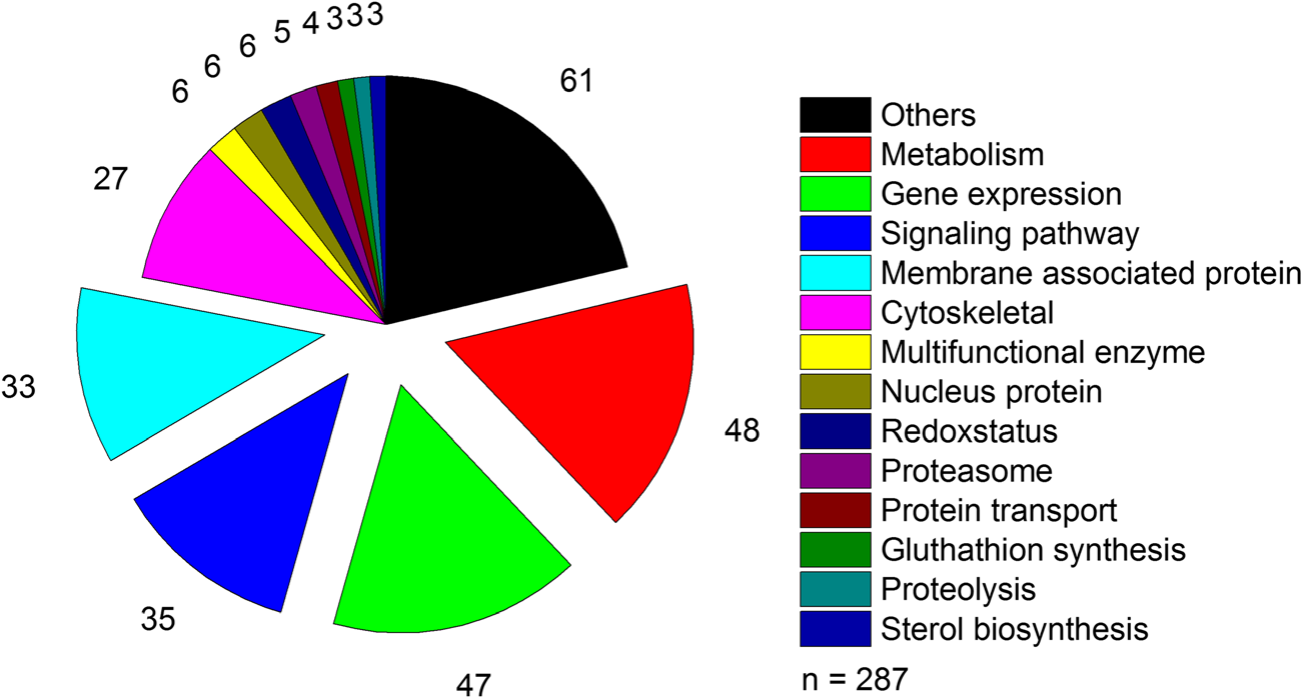
Classification of differentially expressed proteins for MDCK_SUS2_ and MDCK_ADH_ cells. With an induction of at least 1.4-fold, proteins were considered significantly differentially expressed between both cell lines (up and down regulated, *t*-test *p*-value < 0.05, sum of peptide peak intensities of at least three unique peptides, software Progenesis LC-MS). Grouping according to biological processes of human homolog proteins described in Uniprot (Consortium 2014). All details for protein candidates and quantification are given in the Online Resource 3

Beyond MS-based analysis of expression levels of enzymes, a total of 976 proteins was examined for differences in MDCK_SUS2_ and MDCK_ADH_ cell cultivations. Analysis of protein data (Student’s *t*-test with a *p*-value < 0.05, at least 1.4-fold expression) resulted in 287 differentially expressed proteins (Online Resource 3). Most of the proteins could be assigned to the following functional classes: metabolism, gene expression, signaling pathway, and membrane associated protein (Fig. 2). Thus, a comprehensive change in protein expression of MDCK_SUS2_ cells occurred after cell line adaptation with obvious alterations in adhesive cell membrane components and intracellular signaling pathways. To focus the analysis, these 287 proteins were evaluated by Uniprot database search and literature research to identify interconnections to the observed alterations in enzyme activity levels of MDCK_SUS2_ and MDCK_ADH_ cells. Of those, pirin, cadherin-1, caveolin (CAV1), and AMP-activated protein kinase (AMPK) were described to have a direct impact on cellular metabolism (Table 3).

**Table 3:** Proteins with a statistically significant difference in expression for MDCK_SUS2_ and MDCK_ADH_ cells including literature-based links to observed metabolic changes

Protein	Described metabolic change	Increased protein expression	Reference
Pirin	Inhibits pyruvate dehydrogenase activity by expression of a pirin ortholog in *Serratia marcescens*.	MDCK_SUS2_	(Soo et al. 2007)
Cadherin	Reduced glycolytic activity by knock-down in breast cancer cells.	MDCK_ADH_	(Chu et al. 2013)
Caveolin	Is involved in the modulation of glycolytic activity as well as mitochondrial bioenergetics. Colorectal cancer cells resulted in the enhancement of aerobic glycolysis by CAV1 expression. Suppressive impact of CAV1 on mitochondrial activities was described for 3T3NIH fibroblast cells.	MDCK_ADH_	(Nwosu et al. 2016) (Ha et al. 2012) (Rimessi et al. 2014)
AMP-activated protein kinase	Induces a p53-dependent cell proliferation upon glucose availability	MDCK_SUS2_	(Jones et al. 2005)

The list of significantly differentially expressed protein candidates (Online Resource 3) highlighted also various proteins of the respiratory chain (eight proteins) to be increased on average 1.6-fold for MDCK_SUS2_ cells compared to MDCK_ADH_ cells (Table 4). These findings might be related to the expression of CAV1, which is involved in mitochondrial membrane structure as described by Nwosu et al. (2016). Additionally, levels of four Ras-related proteins were decreased for MDCK_SUS2_ cells (Table 4). MDCK cells are widely used for studies of epithelial-mesenchymal transition. In general, MDCK cells show an epithelial phenotype, while oncogenic Ras-transformed MDCK cells undergo a transition and show a mesenchymal phenotype (Shukla et al. 2015). However, the decreased expression of Ras-related proteins for MDCK_SUS2_ cells did not seem to be correlated with the Ras-induced transition pathway.

**Table 4:** Selected proteins with a statistically significant difference in expression for MDCK_SUS2_ and MDCK_ADH_ cells

Protein	Peptides^a^	Ratio MDCK_SUS2_/MDCK_ADH_^b^	*p*-value ^c^
Proteins of respiratory chain			
ATP synthase, H+ transporting, mitochondrial F0 complex, subunit B1	5 (4)	1.41	*
ATP synthase-coupling factor 6, mitochondrial	4 (4)	1.45	***
ATPase family, AAA domain–containing 3A	6 (5)	1.47	***
Cytochrome b-c1 complex subunit 1, mitochondrial	8 (8)	1.57	**
Electron-transfer-flavoprotein, alpha polypeptide	9 (6)	1.74	***
NADH dehydrogenase (ubiquinone) flavoprotein 2, 24 kDa	4 (4)	1.98	***
Similar to cytochrome c-1	4 (3)	1.63	***
Similar to Ubiquinol-cytochrome-c reductase complex core protein 2, mitochondrial precursor (complex III subunit II)	5 (5)	1.44	***
Ras-related proteins			
RAB3D, member Ras oncogene family	4 (3)	0.49	***
Ras GTPase-activating-like protein IQGAP1	36 (33)	0.58	***
Ras-related protein Rab-10	6 (4)	0.59	***
Ras-related protein Rab-21	6 (4)	0.56	***

^a^Shown are peptide amounts detected with mass spectrometry and unique peptides used for protein quantification (in brakets) in the software Progenesis LC-MS

^b^Ratio for peptide intensity values (summed up for peptides for quantification extracted from the software Progenesis LC-MS) comparing MDCK_SUS2_ cells with MDCK_ADH_ cells

^c^Statistical significance of the ratio for peptide intensity (*t*-test, *p*-value (*) < 0.01, (**) < 0.001, (***) < 0.0001)

## Discussion

The complex process of MDCK cell adaptation to growth in suspension in a chemically defined medium was firstly analyzed regarding basic aspects such as general growth properties and metabolism (Lohr et al. 2010). In a proteomic study, differences between the parental and MDCK_SUS2_ cells during stepwise adaptation and the potential function of various proteins involved were investigated (Kluge et al. 2015). In this work, as a new approach, model-based growth characterization, enzyme activity analysis, and in-depths proteomics were combined with the aim to evaluate interconnections and correlations of cellular adaptation processes more holistically and to uncover more general rules of this complex process. A variety of hypotheses are discussed in the following.

The applied cell growth model describes the data of the MDCK_SUS2_ growth in a stirred tank bioreactor well and allows a detailed comparison of suspension and adherent growth for MDCK cells and their basic metabolism. In general, the specific cell growth rates for MDCK_SUS_ were reduced to 30% compared to MDCK_ADH_, which had an influence on the uptake and use of substrates. However, the general metabolic concept of using Glc and Gln during cell growth and shifting to Glu consumption during growth inhibition (Rehberg et al. 2013a) was very consistent with MDCK_ADH_ cells. At the end of cultivations, the cells transitioned to cellular maintenance metabolism that differed in glucose use between both cell lines.

A clear separation of growth phases for MDCK_SUS2_ cells based on cell concentration or cell volume as described for the adherent cell line was difficult (Rehberg et al. 2013a). As expected, an extended lag phase after inoculation, which is due to attachment of microcarriers before initiation of cell division steps, was missing for MDCK_SUS2_ cells compared to MDCK_ADH_ cells. It seems that dynamics of MDCK_SUS2_ cell growth is more susceptible to differences in inoculation conditions. At the end of cultivations, MDCK_ADH_ are usually fully growth inhibited by the limitation in microcarrier surface, while the inhibition of MDCK_SUS2_ cell growth seems to be a related to various factors, i.e., reduced nutrient levels and high cell densities. Apart from growth inhibition, both cell lines are similar in their overall growth behavior. Since nutrient use differed significantly between the cell lines, a shift in intracellular metabolism and a re-routing of cellular building blocks was apparent.

In case of glucose, the volume-specific Glc uptake of MDCK_SUS2_ cells, at the beginning of cultivations, started only at 40 mmol/L/h (Fig. 1) compared to the 200 mmol/L/h of MDCK_ADH_ (Rehberg et al. 2013a). This corresponds to a reduction to 20% in glycolytic activity and correlates with a reduction in cell growth (25–30%) and in measured maximum enzyme activities to 4–40%. Apparently, adaptation of MDCK cells to growth in suspension resulted in a consistent reduction in growth, glucose uptake, and glycolytic enzyme activities. The cell growth specific yield coefficient for glucose also dropped to 20% and the yield coefficient for lactate was reduced to 40%, which means that glucose was used more efficiently for cell growth. The cell volume–specific Gln uptake slightly increased in MDCK_SUS2_ cells to 115%, while maximum enzyme activities that contribute to the citric acid cycle were reduced to 12–85%. Therefore, a correlation between enzyme activities and uptake of Gln was not seen for the citric acid cycle. However, a correlation between uptake of Gln and enzyme protein levels existed. Since the cell growth specific yield coefficient for Gln remained high in MDCK_SUS_ one hypothesis is that Gln use in the citric acid cycle was still high, as explained in the following section. Another hypothesis is that Gln was not fully used in the citric acid cycle, but for other anaplerotic reactions. Therefore, changes in nutrient uptake could be compensated by changes of the corresponding enzyme activities if these nutrients were not required in larger amounts by other pathways.

All measurements for monitoring the maximum enzyme activity were related to the protein amount per cell. Therefore, an increase in maximum enzyme activity may indicate a higher expression of the enzyme relative to other cellular proteins. This implies that a shift in enzyme activity required an increase in expression level of single proteins. Under these assumptions, proteomic analysis correlated with maximum enzyme activity and could explain about 65% of altered enzyme activities of MDCK_SUS2_ cells compared to MDCK_ADH_ cells (15 measured enzymes of 23 detected enzymes by proteomics). Interestingly, protein expression of enzymes of the citric acid cycle was upregulated for MDCK_SUS2_ cells, which fits to the high Gln uptake discussed earlier, but translates not into maximum enzyme activities as a decrease was observed. Higher protein levels but lower maximum enzyme activities may speak for differences in the level of co-factors or differences in protein modifications, which can both modulate the specific enzyme activity. A similar picture is seen for PDH and pyruvate carboxylase and may, again, point towards a control of metabolism at the level of enzyme activity rather than enzyme concentration.

**Fig. 3:**
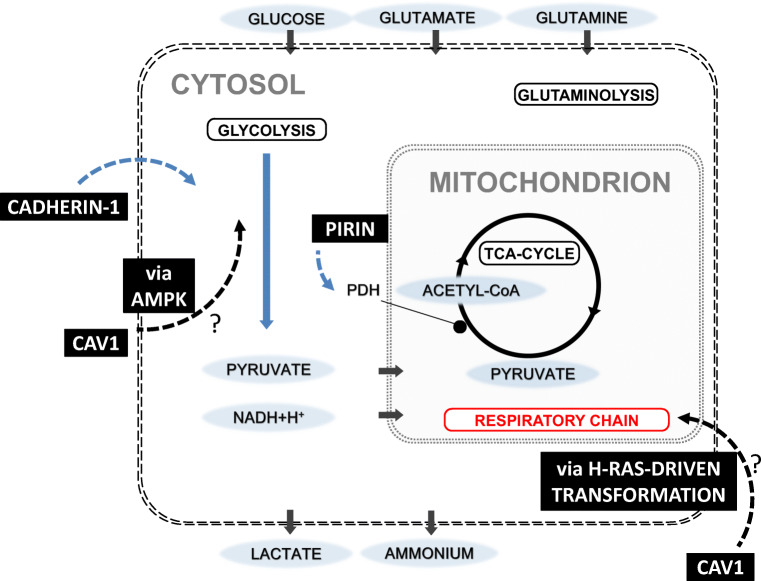
Key players for regulatory mechanisms that could contribute to metabolic adaptations of MDCK cells to growth in suspension (black squares and dashed arrows). Blue, decreased activity or inhibition; red, increased activity. Anaplerotic reactions showed no impact and were not displayed

The evaluation of proteomic data uncovered four regulatory mechanisms that could contribute to metabolic adaptations of the MDCK_SUS2_ cell line (summarized in Fig. 3). First, a 4-fold increase in pirin expression was observed for the suspension cell line. A pirin ortholog was examined in the bacterium *Serratia marcescens*, which bound to the PDH subunit E1 and inhibited PDH enzyme complex activities (Soo et al. 2007). A similar mode of action could be assumed for MDCK_SUS2_ cells with pirin decrease in maximum enzyme activity despite the increase in the protein expression level of PDH (Table 2).

Second, the cell-cell adhesion protein cadherin-1 (also known as E-cadherin) was distinctly less expressed in MDCK_SUS2_ cells (Online Resource 3), certainly, related to loss of adhesive connections after adaptation to suspension growth. Moreover, a study with tumor cells of breast cancer showed a direct correlation of decreased E-cadherin expression and decreased glycolytic capacity mediated by the loss of hypoxia response genes (Chu et al. 2013).

Third, several studies support that CAV1 is involved in the modulation of glycolytic activities as well as bioenergetics of mitochondria, as summarized by Nwosu et al. (2016). However, the mechanisms for the CAV1-mediated regulation of glycolysis and mitochondrial function are largely unclear. A detailed study on colorectal cancer cells described the enhancement of aerobic glycolysis by increased CAV1 expression (Ha et al. 2012). In this study, MDCK_SUS2_ cells showed a down-regulation of glycolytic protein expression (Table 2), which could be related to the reduced expression levels of CAV1. The modulation mechanism of glycolysis activity by CAV1 was described to be related to AMPK activation followed by a p53-dependent G1 cell cycle arrest and autophagy (Ha et al. 2012). Assuming a similar mechanism for MDCK cells, AMPK was evaluated and found to be increased 4-fold for MDCK_SUS2_ cells compared to MDCK_ADH_ cells (Table 3). AMPK is further described to be an important metabolic checkpoint which regulates cell cycle arrest by p53 in mammalian cells (Jones et al. 2005) and its role should be further investigated in more detailed metabolic studies for MDCK cells.

Fourth, regarding the obvious shift in glycolytic enzyme activity, a total of eight respiratory chain proteins were detected to be on average 1.6-fold increased for MDCK_SUS2_ cells compared to MDCK_ADH_ cells (Table 4). An increased protein expression of respiratory chain enzymes is adding a further option for a shift in intracellular metabolism. This cellular adaptation could balance the demand of cellular energy. Among many other possibilities, these proteomic findings might also be related to the reduced expression of CAV1 as already mentioned. Inhibition of mitochondrial activities by CAV1 was described to be related to mediate a suppressor activity in H-Ras–driven transformation (Rimessi et al. 2014). Correspondingly, when evaluating Ras-related proteins in our experimental set-up, it could be seen that four Ras-related proteins had a decreased expression level in MDCK_SUS2_ cells (Table 4).

With the chosen approach in this study, interconnections and correlations on different cellular levels were tracked and key players were identified regarding the adaptation of MDCK cells to growth in suspension. Clearly, by combining the findings of complex analytics and mathematical model evaluation allowed for deeper insights into the cellular response after cell line adaptation. Based on our approach, next steps in verifying the role of possible key players in animal cell metabolism should apply multi-disciplinary approaches that combine in-depth metabolite and enzyme analytics and proteomics together with mathematical modeling to capture the full network of interactions within a cell and its adaptations to new conditions.

## Supplementary Information

ESM 1 (PDF 408 kb)

ESM 2 (PDF 60.1 kb)

## Data Availability

All relevant data and material is given in the manuscript.
