# Supplementary material for: Tracking changes in adaptation to suspension growth for MDCK cells: cell growth correlates with levels of metabolites, enzymes and proteins
Source: Appl Microbiol Biotechnol. 2021 Feb 13;105(5):1861–74. doi: 10.1007/s00253-021-11150-z (PMC7907048; doi:10.1007/s00253-021-11150-z)
Supplement: Supplementary file 1 — (PDF 408 kb) [file 253_2021_11150_MOESM1_ESM.pdf]

Applied Microbiology and Biotechnology

Tracking changes in adaptation to suspension growth for MDCK cells: Cell growth correlates with levels of metabolites, enzymes and proteins

Sabine Pech[a,1], Markus Rehberg[b,1], Robert Janke[b], Dirk Benndorf[a], Yvonne Genzel[b,*], Thilo Muth[c], Albert Sickmann[d,e,f], Erdmann Rapp[b,g], Udo Reichl[a,b]

[a]Otto von Guericke University Magdeburg, Bioprocess Engineering, Magdeburg, Germany

[b]Max Planck Institute for Dynamics of Complex Technical Systems, Bioprocess Engineering, Magdeburg, Germany

[c]Section S.3 eScience, Federal Institute for Materials Research and Testing (BAM), Berlin, Germany

[d]Leibniz-Institut für Analytische Wissenschaften – ISAS – e.V., Dortmund, Germany

[e]Medizinische Fakultät, Medizinisches Proteom-Center (MPC), Ruhr-Universität Bochum, Bochum, Germany

[f]Department of Chemistry, College of Physical Sciences, University of Aberdeen, Aberdeen, Scotland, UK

[g]glyxera GmbH, Magdeburg, Germany

[1]shared first authorship

[*]corresponding author to: genzel@mpi-magdeburg.mpg.de
ORCID: 0000-0002-2652-5943

Online Resource 1

Caption: Estimated initial conditions and parameters for each experiment during model optimization as well as respective pre-culture conditions. Symboles Δ, □ and ○ referring to Figure 1. Data of the two independent experiments for enzyme activity measurements indicated as triangle (Δ) and rectangle (□). Data of the three independent experiments for proteomic analysis with minimal sampling were combined and average is indicated as circle (○).

local parameter	experiment Δ	experiment □	experiment ○	unit
$X_{tot}(t = 0)$	1.84×10^6	2.92×10^6	1.40×10^6	cells mL
$[Glc](t = 0)$	21.38	21.34	17.33	mmol L^{-1}
$[Gln](t = 0)$	7.69	7.84	3.34	mmol L^{-1}
$[Glu](t = 0)$	2.89	3.46	1.53	mmol L^{-1}
$[Lac](t = 0)$	0.00	0.00	0.16	mmol L^{-1}
$[NH4](t = 0)$	0.00	0.00	0.00	mmol L^{-1}
d_m	11.04	11.76	11.47	µm
d_c	16.00	14.40	14.20	µm

Online Resource 2

Caption: Air flow rates of the bioreactor cultivations (cellferm-pro® system, DasGip AG, Germany) of $MDCK_{ADH}$ and $MDCK_{SUS2}$ cells. Air flow rates were controlled by an oxygen probe (set-point: dissolved oxygen 40%, reference: air saturation) and an approximated estimation for the oxygen demand is possible. The comparative assessment is based on the slope of an exponential regression calculated with Excel (considert data points from 12 h cultivation time). Data from the third replicate of $MDCK_{ADH}$ were excluded due to strong variations in measurements of the oxgen probe. $MDCK_{ADH}$ – 2 replicates colored orange, regression: $y = 0.4293e^{0.1877x}$ and pink, regression: $y = 0.3849e^{0.2203x}$, $MDCK_{SUS2}$ – 3 replicates colored green, regression: $y = 0.4125e^{0.1589x}$, dark blue, regression: $y = 0.5653e^{0.0798x}$ and light blue, regression: $y = 0.4244e^{0.0995x}$); sL – standard liter (at 0°C = 273 K and 1013 mbar).

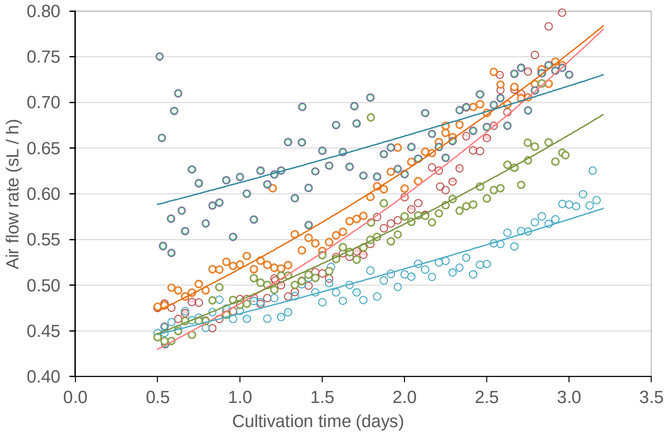

Online Resource 3

Caption: Protein candidates and quantification which resulted in a statistically significant difference in expression for $MDCK_{SUS2}$ and $MDCK_{ADH}$ cells. Shown are peptide amounts detected with mass spectrometry and unique peptides used for protein quantification (in brakets) in the software Progenesis LC-MS.

[a]Statistical significance of the ratio for peptide intensity calculated with R project (t-Test, p-value).

[b]Ratio for peptide intensity values (summed up for peptides for quantification extracted from the software Progenesis LC-MS) comparing $MDCK_{SUS2}$ cells with $MDCK_{ADH}$ cells.

Classification	Protein name	Peptide count	Peptides used for quantitation	Confidence search score	p-value[a]	Accession	Ratio[b] $MDCK_{SUS2}/MDCK_{ADH}$
Cytoskeletal	Actin, aortic smooth muscle	14	4	1.70E+03	2.20E-04	F2Z4N0;*	0.548
Cytoskeletal	Actin-binding protein anillin	13	12	1.46E+03	2.20E-06	F1PZ90	0.553
Cytoskeletal	Actin-related protein 2/3 complex subunit 1B	9	9	1.20E+03	2.90E-05	E2RMT4	0.639
Cytoskeletal	ARP2 actin-related protein 2 homolog (yeast)	8	3	8.98E+02	3.40E-07	E2QXY7	0.649
Cytoskeletal	Calcium/calmodulin-dependent protein kinase type II subunit delta	7	6	7.00E+02	1.40E-07	E2RR74*	1.826
Cytoskeletal	capping protein (actin filament), gelsolin-like	9	6	1.00E+03	5.80E-07	E2R413	0.450
Cytoskeletal	chloride intracellular channel 4	11	8	1.70E+03	1.90E-07	E2RGI4*	2.144
Cytoskeletal	Collagen alpha-2(V) chain	23	17	2.47E+03	3.20E-06	F1PG08	2.532
Cytoskeletal	Coronin-1C	4	4	4.00E+02	1.10E-02	F1Q1F6*	0.580
Cytoskeletal	Cytoskeleton-associated protein 4	22	15	2.79E+03	3.50E-05	F1PEI2	1.436
Cytoskeletal	Keratin, type I cytoskeletal 19	23	11	2.58E+03	2.90E-07	F1Q0N9	0.296
Cytoskeletal	Keratin, type II cytoskeletal 7	13	6	1.27E+03	1.10E-07	F1PRB0	0.510
Cytoskeletal	La ribonucleoprotein domain family, member 4	6	6	5.95E+02	1.60E-06	E2R028*	0.674
Cytoskeletal	LIM domain and actin binding 1	20	16	2.50E+03	7.40E-06	E2QW54	0.693
Cytoskeletal	PDZ and LIM domain 5	10	8	1.00E+03	1.90E-15	E2RBA8	0.177
Cytoskeletal	Periplakin	23	19	2.49E+03	4.50E-12	J9NU37	0.321
Cytoskeletal	phosphohistidine phosphatase 1	3	3	4.00E+02	6.60E-06	E2R1M6	2.150
Cytoskeletal	Profilin 1	5	4	6.00E+02	6.00E-07	F1Q3Y0	0.465
Cytoskeletal	similar to crystallin, zeta	7	6	8.85E+02	5.50E-08	E2R3I8	2.394
Cytoskeletal	Spectrin alpha chain, non-erythrocytic 1	83	76	1.03E+04	2.10E-11	J9P2H0	0.602
Cytoskeletal	tropomodulin 3 (ubiquitous)	6	6	6.98E+02	8.90E-05	E2QWL1	0.675
Cytoskeletal	tubulin folding cofactor B	4	4	4.00E+02	4.90E-06	E2QYN0	0.524
Cytoskeletal	Tubulin-specific chaperone A	5	4	5.93E+02	2.90E-02	F1PVY1*	1.411
Cytoskeletal	Twinfilin-1	6	4	7.00E+02	3.20E-06	J9NRP8	0.447
Cytoskeletal	Vacuole membrane protein 1	3	3	3.00E+02	7.60E-06	F1PHP3	2.169
Cytoskeletal	Villin-1	14	13	1.60E+03	3.20E-09	E2RPX1	3.822
Cytoskeletal	Vimentin	42	34	5.36E+03	1.30E-08	F1PLS4	0.313
Gene expression	60S ribosomal protein L5	11	6	1.30E+03	6.90E-05	F1P7B0	1.414
Gene expression	60S ribosomal protein L7	9	7	1.30E+03	3.60E-06	F1Q0Z2	1.547

Gene expression	BCL2-associated transcription factor 1	10	9	1.40E+03	1.20E-04	E2R2T1*	1.418
Gene expression	cofactor of BRCA1	3	3	3.98E+02	1.80E-02	E2QX07	1.422
Gene expression	cytochrome P450, family 1, subfamily B, polypeptide 1	6	5	7.00E+02	1.00E-05	C1KG39*	1.961
Gene expression	Developmentally-regulated GTP-binding protein 1	5	4	5.00E+02	5.40E-05	J9P0A6	0.543
Gene expression	Echinoderm microtubule-associated protein-like 4	15	11	2.08E+03	1.50E-05	F1PY01	1.435
Gene expression	Elongation factor 1-beta	4	3	4.00E+02	2.60E-06	J9NT16	0.535
Gene expression	Eukaryotic translation initiation factor 3 subunit A	22	22	2.48E+03	7.30E-08	E2RSR5	0.646
Gene expression	Eukaryotic translation initiation factor 3 subunit B	14	13	1.70E+03	2.30E-09	F1P969	0.590
Gene expression	eukaryotic translation initiation factor 3, subunit G	6	6	6.00E+02	9.50E-04	E2RGG6	0.699
Gene expression	Eukaryotic translation initiation factor 4B	9	9	1.08E+03	1.20E-07	E2RKS4	0.440
Gene expression	GATA zinc finger domain containing 2B	5	4	5.00E+02	4.30E-04	E2RFR6*	1.774
Gene expression	heterogeneous nuclear ribonucleoprotein D-like	7	6	6.97E+02	1.70E-03	E2QRT0	0.666
Gene expression	Heterogeneous nuclear ribonucleoproteins A2/B1	20	18	2.97E+03	1.20E-05	F1Q1K6	1.576
Gene expression	High mobility group protein B3	4	4	3.99E+02	4.10E-05	J9NVS3	1.446
Gene expression	high-mobility group box 2	11	7	1.20E+03	3.00E-08	E2QY30	2.576
Gene expression	Insulin-like growth factor 2 mRNA-binding protein 1	12	9	1.29E+03	8.50E-10	E2RTC5	0.234
Gene expression	Lysine--tRNA ligase	7	7	6.95E+02	5.30E-08	E2RSP4	0.575
Gene expression	Myosin-14	36	21	3.98E+03	3.40E-05	J9NU82	1.470
Gene expression	NOP56 ribonucleoprotein homolog (yeast)	15	11	1.60E+03	8.90E-05	E2QU53	1.509
Gene expression	Polymerase I and transcript release factor	8	8	8.97E+02	5.70E-08	J9P923	0.441
Gene expression	polyribonucleotide nucleotidyltransferase 1	14	12	1.50E+03	3.90E-06	E2QWK2	1.726

Gene expression	Pre-mRNA-processing factor 19	9	7	1.40E+03	1.10E-04	E2R8L3	1.408
Gene expression	Probable tRNA N6-adenosine threonylcarbamoyltransferase	4	4	4.00E+02	2.60E-06	J9P2K2	1.845
Gene expression	protein arginine methyltransferase 5	6	5	7.96E+02	1.60E-04	E2R9J8	1.743
Gene expression	Ribonuclease UK114	3	3	4.71E+02	1.60E-05	E2QS94	1.604
Gene expression	ribosomal protein 17-like; similar to 60S ribosomal protein L17 (L23); ribosomal protein L17	8	6	9.95E+02	9.70E-05	J9NUV8*	1.587
Gene expression	ribosomal protein L15	4	4	3.98E+02	1.50E-06	E2QXF3	1.762
Gene expression	ribosomal protein L8	3	3	5.00E+02	4.20E-05	E2RIA8	1.435
Gene expression	Ribosomal protein S3A; similar to ribosomal protein S3a	9	8	8.97E+02	3.70E-04	F2Z4Q5*	1.408
Gene expression	RNA (guanine-7-) methyltransferase	8	6	1.00E+03	4.10E-05	E2R4R3	1.447
Gene expression	RNA-binding protein FUS isoform 1	6	4	9.00E+02	7.00E-08	F1PBJ4	0.633
Gene expression	seryl-tRNA synthetase	11	10	1.30E+03	1.10E-03	E2R4D5	0.477
Gene expression	Signal recognition particle subunit SRP68	10	6	1.19E+03	2.70E-04	F1PI42*	1.412
Gene expression	similar to H2A histone family, member Y isoform 3	6	6	6.99E+02	3.50E-08	E2RCZ6*	1.549
Gene expression	similar to Heterogeneous nuclear ribonucleoprotein A1	16	14	2.80E+03	1.20E-05	E2QYP1	0.559
Gene expression	similar to Putative eukaryotic translation initiation factor 3 subunit (eIF-3)	7	5	7.00E+02	1.50E-03	E2R9L7	1.473
Gene expression	similar to ribosomal protein L18; ribosomal protein L18	4	4	3.99E+02	4.90E-04	A1DZY5	2.008
Gene expression	similar to ribosomal protein S24 isoform 3; similar to ribosomal protein S24	3	3	4.00E+02	2.20E-03	J9PAP2*	0.622
Gene expression	similar to Splicing factor 3A subunit 3 (Spliceosome associated protein 61) (SAP 61) (SF3a60); similar to splicing factor 3a, subunit 3 (predicted)	6	5	6.97E+02	8.00E-03	E2QXU5	0.398
Gene expression	Splicing factor 1	7	7	9.99E+02	2.80E-04	E2RRT4	0.694
Gene expression	tankyrase 1 binding protein 1, 182kDa	21	17	2.37E+03	3.20E-09	E2R6L1	0.581
Gene	transformer 2 beta homolog (Drosophila)	3	3	3.00E+02	4.20E-03	E2RD37	0.700

expression							
Gene expression	transportin 3	12	11	1.20E+03	1.20E-03	E2REK5	0.557
Gene expression	tyrosyl-tRNA synthetase	9	8	8.97E+02	1.60E-04	E2RHR7	1.675
Gene expression	U4/U6.U5 tri-snRNP associated protein 1	8	6	8.79E+02	6.90E-05	F1PQU2	1.610
Gluthathion synthesis	CNDP dipeptidase 2 (metallopeptidase M20 family)	12	10	1.50E+03	7.10E-07	E2R134	1.683
Gluthathion synthesis	gamma-glutamyl cyclotransferase	6	6	6.00E+02	9.50E-04	E2R9K5	1.433
Gluthathion synthesis	Glutamate--cysteine ligase catalytic subunit	8	8	8.98E+02	1.20E-08	E2RPJ4	4.633
Membrane associated protein	actinin, alpha 1	29	13	3.59E+03	6.00E-06	E2QY07*	0.669
Membrane associated protein	Agrin	14	11	1.50E+03	2.30E-06	F1Q2Z6	0.438
Membrane associated protein	Annexin A1; Annexin	6	6	6.00E+02	6.60E-10	F1PTI7	2.160
Membrane associated protein	Annexin A13	11	8	1.38E+03	8.60E-07	J9P497*	3.127
Membrane associated protein	Annexin A5	7	6	8.83E+02	2.30E-03	E2RQ14	1.590
Membrane associated protein	annexin A8-like 1	9	6	1.10E+03	4.70E-04	E2R0S6	0.629
Membrane associated protein	AT-rich interactive domain-containing protein 1A	5	5	4.98E+02	9.00E-03	E2RSH9	1.479
Membrane associated protein	Cadherin-1	4	3	4.00E+02	1.70E-03	F1PAA4*	0.647
Membrane associated protein	Carnitine O-palmitoyltransferase 1, liver isoform	9	7	1.00E+03	1.90E-03	F1PM56	1.423
Membrane associated	Caveolin-1	3	3	4.97E+02	3.20E-07	P33724	0.514

protein							
Membrane associated protein	Desmoglein-2	15	9	1.70E+03	3.90E-07	F1PGX1*	0.427
Membrane associated protein	Ephrin type-A receptor 1	4	4	4.00E+02	2.00E-07	E2R6A6	0.221
Membrane associated protein	Epididymis luminal protein 57 (Homo sapiens)	6	6	5.99E+02	3.90E-06	E2RKJ6	0.426
Membrane associated protein	Galectin-1	4	4	5.00E+02	5.70E-12	E2RJL1*	0.095
Membrane associated protein	Galectin-3	10	9	1.48E+03	3.80E-09	E5Q8W5*	0.524
Membrane associated protein	Integrin alpha-6	4	3	4.00E+02	9.80E-03	E2RL88*	0.401
Membrane associated protein	Integrin beta-3	7	7	6.99E+02	1.40E-07	F1PPG5*	0.643
Membrane associated protein	Isoform Beta-4A of Integrin beta-4	10	9	1.10E+03	3.90E-10	J9NXR3	0.470
Membrane associated protein	L1 cell adhesion molecule	12	8	1.20E+03	9.60E-09	E2R2V5*	0.485
Membrane associated protein	moesin	20	10	2.20E+03	1.40E-07	E2R7F1	0.370
Membrane associated protein	Myoferlin	20	15	2.20E+03	1.20E-09	F1PZD5*	0.469
Membrane associated protein	myosin VI	22	19	2.30E+03	5.10E-07	E2QUR8	1.428
Membrane associated protein	Neuroblast differentiation-associated protein AHNAK	137	107	1.58E+04	9.80E-09	J9P969	0.666
Membrane associated	Plakoglobin	7	5	7.98E+02	6.20E-06	Q6VAH9	0.580

protein							
Membrane associated protein	Protein AHNAK2	15	13	1.89E+03	3.50E-11	J9NZV9	0.317
Membrane associated protein	secreted phosphoprotein 1	11	6	1.50E+03	1.00E-05	E2R161*	0.119
Membrane associated protein	Serpin B6	8	7	1.00E+03	1.40E-03	E2RGP2	0.669
Membrane associated protein	serpin peptidase inhibitor, clade H (heat shock protein 47), member 1, (collagen binding protein 1)	16	12	2.20E+03	4.20E-09	C7C419*	0.564
Membrane associated protein	stomatin	7	5	7.00E+02	1.20E-05	B6F250*	1.685
Membrane associated protein	Syndecan-4; Syndecan	4	3	4.97E+02	1.20E-05	J9NZ79	0.421
Membrane associated protein	Syntenin-1	13	13	1.70E+03	5.00E-05	F1PKR0	0.610
Membrane associated protein	Thrombospondin-1	13	11	1.59E+03	1.40E-08	F1PBI6	2.573
Membrane associated protein	Utrophin	19	13	2.00E+03	1.90E-10	J9P1H1*	0.351
Metabolism	3-hydroxyisobutyrate dehydrogenase, mitochondrial	12	8	1.20E+03	5.40E-06	E2QXD5*	1.893
Metabolism	**6-phosphofructokinase, liver type**	11	7	1.30E+03	6.20E-05	F1Q3S9	0.634
Metabolism	Acetyl-CoA acetyltransferase, cytosolic	5	5	7.00E+02	8.50E-04	F1Q466	0.437
Metabolism	**Acetyl-coenzyme A synthetase 2-like, mitochondrial**	3	3	3.00E+02	1.00E-03	F1PUA2	1.544
Metabolism	Acid ceramidase	4	3	3.98E+02	1.00E-09	F1PWA9	0.225
Metabolism	**aconitase 2, mitochondrial**	20	14	2.30E+03	7.90E-04	E2RCY8	1.467
Metabolism	acyl-CoA synthetase long-chain family member 1	13	10	1.29E+03	4.90E-09	E2QUL3	2.175
Metabolism	Acyl-coenzyme A thioesterase 9, mitochondrial	4	4	4.89E+02	5.40E-05	E2RMR8*	0.431
Metabolism	ATP synthase, H+ transporting, mitochondrial F0 complex, subunit B1	5	4	5.97E+02	4.20E-03	E2QW06	1.406
Metabolism	ATP synthase-coupling factor 6, mitochondrial	4	4	6.00E+02	5.80E-06	E2RL03	1.447

Metabolism	ATPase family, AAA domain containing 3A	6	5	6.98E+02	9.60E-05	E2QZ61	1.467
Others	copine III	5	5	5.99E+02	1.10E-03	E2QZQ6*	0.475
Metabolism	cystathionase (cystathionine gamma-lyase)	5	4	5.98E+02	5.50E-07	E2RDY8	0.566
Metabolism	Cytochrome b-c1 complex subunit 1, mitochondrial	8	8	1.10E+03	5.70E-04	E2RSI5	1.565
Metabolism	Cytoplasmic aconitate hydratase	7	5	8.00E+02	8.80E-04	E2RMX9*	0.575
Metabolism	Delta-1-pyrroline-5-carboxylate dehydrogenase, mitochondrial	7	4	7.78E+02	2.90E-05	E2QZP8*	1.536
Metabolism	Deoxyuridine 5'-triphosphate nucleotidohydrolase, mitochondrial (Homo sapiens)	3	3	3.00E+02	4.30E-04	E2RRF8	0.449
Metabolism	electron-transfer-flavoprotein, alpha polypeptide	9	6	1.20E+03	2.80E-05	E2RAE2	1.741
Metabolism	Fatty acid synthase (Homo sapiens)	63	54	7.59E+03	5.10E-07	F1Q2F6	1.425
Metabolism	Flap endonuclease 1	3	3	3.00E+02	1.70E-03	J9PB88	1.618
Metabolism	**Fructose-bisphosphate aldolase A**	28	17	3.59E+03	3.20E-06	F1PBT3	0.547
Metabolism	**fumarate hydratase**	7	5	8.00E+02	1.10E-03	E2RGR9	1.466
Others	Glucosidase 2 subunit beta	6	5	6.00E+02	4.60E-06	E2RKK6	0.698
Metabolism	**Glutaminase kidney isoform, mitochondrial**	5	5	6.96E+02	2.60E-04	E2RJ93	0.510
Metabolism	glycerol-3-phosphate dehydrogenase 2 (mitochondrial)	8	7	7.97E+02	9.40E-05	E2RAZ8	0.459
Metabolism	Golgi membrane protein 1	3	3	4.00E+02	1.30E-08	E2RLA5	0.158
Metabolism	**Hexokinase-2**	10	4	1.00E+03	4.70E-06	F1PAZ2	0.596
Metabolism	Hypoxanthine-guanine phosphoribosyltransferase	8	6	8.00E+02	1.10E-06	J9NVT2	2.188
Metabolism	**Isocitrate dehydrogenase [NADP], mitochondrial; Isocitrate dehydrogenase [NADP]**	6	6	7.00E+02	2.10E-05	P49819	1.757
Metabolism	**L-lactate dehydrogenase A chain**	18	17	2.49E+03	2.20E-04	F1PVW0	0.585
Metabolism	Long-chain-fatty-acid--CoA ligase 4	12	8	1.20E+03	2.00E-07	F1PEH9	0.473
Metabolism	Methylmalonate-semialdehyde dehydrogenase [acylating], mitochondrial	7	4	6.97E+02	4.30E-06	J9P2I9	1.912
Metabolism	NADH dehydrogenase (ubiquinone) flavoprotein 2, 24kDa	4	4	3.99E+02	1.30E-06	E2QVP4	1.975
Metabolism	NADH-cytochrome b5 reductase 3	10	7	1.20E+03	9.80E-06	F1PS26*	0.258
Metabolism	**NADP-dependent malic enzyme; Malic enzyme**	9	7	1.17E+03	8.50E-08	J9NZX7	0.237
Metabolism	oxysterol binding protein-like 3	15	12	1.70E+03	2.60E-10	E2QYC5*	0.578
Metabolism	**Phosphoglycerate kinase; Phosphoglycerate kinase 1**	13	12	1.60E+03	7.90E-07	E2RRC9	0.450
Metabolism	Pirin	4	4	5.00E+02	3.50E-07	E2QSR0*	4.335
Metabolism	Purine nucleoside phosphorylase	9	9	1.30E+03	2.40E-06	F1PQM1	0.607

Metabolism	**Pyruvate carboxylase, mitochondrial**	6	4	7.00E+02	3.10E-04	F1P6G9	1.631
Metabolism	**Pyruvate dehydrogenase (lipoamide) beta**	8	5	9.99E+02	2.70E-04	J9P208*	1.456
Metabolism	**Pyruvate kinase; Pyruvate kinase PKM**	35	30	5.00E+03	1.60E-06	F1PHR2	0.604
Metabolism	similar to Adenosylhomocysteinase (S-adenosyl-L-homocysteine hydrolase) (AdoHcyase); adenosylhomocysteinase	9	7	1.10E+03	2.70E-05	E2QXS7	1.749
Metabolism	similar to cytochrome c-1	4	3	5.00E+02	1.40E-05	E2REM0	1.628
Metabolism	**similar to Glyceraldehyde-3-phosphate dehydrogenase (GAPDH)**	31	16	3.68E+03	9.20E-06	E2RNTJ4	0.667
Metabolism	similar to Ubiquinol-cytochrome-c reductase complex core protein 2, mitochondrial precursor (Complex III subunit II)	5	5	5.00E+02	6.30E-05	E2RHL0	1.436
Metabolism	**Succinate dehydrogenase [ubiquinone] flavoprotein subunit, mitochondrial**	12	9	1.39E+03	6.10E-06	F1Q1X9	1.609
Metabolism	Succinyl-CoA:3-ketoacid coenzyme A transferase 1, mitochondrial	6	5	6.96E+02	4.50E-03	F1PJ13	1.613
Multifunctiona l enzyme	2',3'-cyclic-nucleotide 3'-phosphodiesterase	9	8	1.40E+03	8.60E-06	J9P9V0	2.164
Multifunctiona l enzyme	Bifunctional ATP-dependent dihydroxyacetone kinase/FAD-AMP lyase (cyclizing)	10	8	1.10E+03	5.60E-09	F1Q0K5	2.381
Multifunctiona l enzyme	Calreticulin	13	11	2.09E+03	6.50E-07	E2QU37*	0.549
Multifunctiona l enzyme	Nucleolar GTP-binding protein 1	5	4	5.00E+02	1.00E-05	F1PJY9	1.527
Multifunctiona l enzyme	Retinal dehydrogenase 1	30	4	4.30E+03	9.70E-07	J9P9J4	4.017
Multifunctiona l enzyme	similar to poly(rC) binding protein 2 (predicted); poly(rC) binding protein 2	11	3	1.38E+03	8.40E-03	E2RCF9	0.592
Nucleus protein	importin 5	22	15	2.50E+03	3.40E-03	E2RF06	0.678
Nucleus protein	Importin-7 (Homo sapiens)	10	7	1.19E+03	4.30E-07	E2R2C4*	0.671
Nucleus protein	Lamin-B2	8	6	8.86E+02	6.70E-06	F1Q1E1	2.007
Nucleus protein	Nuclear pore complex protein Nup153	6	5	6.96E+02	3.70E-05	E2RLN2*	0.635
Nucleus protein	nucleoporin 62kDa	5	5	6.00E+02	3.40E-05	E2R2P0	0.579
Nucleus protein	Omega-amidase NIT2	6	4	6.00E+02	1.60E-04	F1PTD1	1.888
Others	-	12	9	1.60E+03	9.00E-06	E2QZM1	0.576

Others	1,4-alpha-glucan-branching enzyme	5	5	4.99E+02	3.50E-08	F1PX32	0.543
Metabolism	3'-phosphoadenosine 5'-phosphosulfate synthase 1	5	5	5.99E+02	5.40E-06	E2QZ13	0.686
Others	Alpha-2-macroglobulin receptor-associated protein	3	3	3.00E+02	7.00E-07	F1PAG7	2.136
Others	Aspartyl/asparaginyl beta-hydroxylase (Bos taurus)	4	3	4.00E+02	1.20E-03	F1Q257;*	0.682
Others	Ataxin-2-like protein	5	3	5.00E+02	6.40E-04	F1PRY9*	1.478
Others	ATP-binding cassette sub-family F member 2	4	4	5.00E+02	1.20E-03	E2RLB4	0.663
Others	Brain-specific angiogenesis inhibitor 1-associated protein 2	5	4	5.00E+02	1.20E-04	F1PL36*	1.796
Others	calcium binding protein 39	4	4	4.99E+02	1.20E-03	E2R8W7	1.489
Others	Carbonic anhydrase 2	11	10	1.48E+03	4.10E-10	F1PDY8	0.350
Metabolism	Carbonyl reductase [NADPH] 1	6	3	6.00E+02	3.80E-05	J9P7P2	1.555
Others	cathepsin B	4	4	5.00E+02	1.50E-05	E2R6Q7	0.498
Others	Cathepsin D (Lysosomal aspartyl peptidase), isoform CRA_a	7	6	7.99E+02	3.10E-06	Q4LAL9	0.440
Others	cDNA FLJ61463, highly similar to Zyxin (Homo sapiens)	7	7	8.97E+02	5.40E-10	F1P7F8	0.377
Others	Cold shock domain-containing protein E1	17	16	2.20E+03	9.50E-08	F1PG95	0.564
Others	Drebrin	4	3	5.00E+02	2.40E-07	J9NXT9	0.654
Others	Dynamin-2	8	5	7.99E+02	6.60E-07	E2RNC3	0.539
Others	Endonuclease domain-containing 1 protein	4	4	5.00E+02	1.60E-08	F1PQA0	0.446
Others	ERO1-like protein alpha	7	5	6.81E+02	1.50E-07	E2RNW5	0.483
Others	Estradiol 17-beta-dehydrogenase 12	7	6	9.97E+02	1.70E-06	E2RJ79	1.817
Others	FERM, RhoGEF (ARHGEF) and pleckstrin domain protein 1 (chondrocyte-derived)	7	7	7.00E+02	9.80E-05	E2REU8	1.739
Others	Ferritin heavy chain	5	3	8.00E+02	3.90E-02	Q95MP7	0.670
Others	FK506 binding protein 10, 65 kDa	14	9	1.58E+03	5.70E-07	E2QZA8	0.573
Others	Glutamine--fructose-6-phosphate aminotransferase [isomerizing] 1	9	7	8.98E+02	7.60E-04	F1PY49	1.519
Others	HCLS1-binding protein 3	4	4	5.00E+02	1.90E-02	E2RT33*	0.662
Others	HLA class II histocompatibility antigen, DR alpha chain	3	3	4.00E+02	7.20E-04	Q30437*	5.796
Others	hypothetical gene supported by AY195837	24	8	3.39E+03	3.10E-03	J9NRT6	0.408
Others	Inverted formin-2 (Homo sapiens)	7	6	8.00E+02	3.10E-10	F1PU24	0.308
Others	Isoform 2 of Sequestosome-1 (Homo sapiens)	5	4	5.00E+02	2.10E-06	F1P6L5	0.218
Others	Isoform Short of Antigen KI-67	15	10	1.47E+03	8.40E-06	E2RJX3	0.670

Others	LDLR chaperone MESD (Bos taurus)	4	4	4.00E+02	1.60E-03	J9P7R9	0.547
Others	Leukotriene A-4 hydrolase	10	9	1.20E+03	3.20E-08	E2QVX4	0.534
Others	Major vault protein	15	13	1.60E+03	2.00E-04	F1P914	0.661
Others	mannose-6-phosphate receptor (cation dependent)	4	4	3.96E+02	1.70E-05	E2R4C1	3.035
Others	Membrane-associated progesterone receptor component 1	4	4	4.98E+02	3.20E-06	E2RJW8	1.908
Others	Mitochondrial import receptor subunit TOM70	5	5	5.00E+02	8.80E-12	E2RKG3	1.975
Others	Mucin-13 (Homo sapiens)	6	5	7.00E+02	7.70E-08	J9P9I4*	5.108
Others	Multifunctional protein ADE2	13	11	1.40E+03	1.80E-03	F1PPT7	1.440
Others	non-metastatic cells 2, protein (NM23B) expressed in	14	8	1.99E+03	3.90E-10	E2RC20*	1.605
Others	phosphatidylinositol binding clathrin assembly protein	4	4	5.00E+02	2.00E-02	E2RCL7*	0.616
Others	phosphoribosyl pyrophosphate synthetase 2	3	3	3.00E+02	4.20E-04	E2R0X3	1.435
Others	Protein CDV3 homolog (Homo sapiens)	4	4	4.98E+02	3.20E-06	F1PZS5	0.648
Others	protein disulfide isomerase family A, member 4	28	25	3.49E+03	7.60E-09	E2R7L1	1.481
Others	Protein FAM114A2	12	7	1.20E+03	2.40E-04	E2RNE4	0.602
Others	Protein kinase C delta-binding protein	3	3	3.00E+02	1.10E-09	F1Q0L3	0.321
Others	protein kinase, cAMP-dependent, regulatory, type I, alpha (tissue specific extinguisher 1)	7	5	8.98E+02	8.90E-07	E2QZV5	4.308
Others	Protein LYRIC	8	6	8.00E+02	1.80E-07	J9P530	1.560
Others	reticulon 4	6	6	8.00E+02	7.10E-08	E2R925*	0.481
Others	Serum albumin	4	3	4.00E+02	2.40E-04	F2Z4Q6*	0.015
Others	similar to CG32066-PB, isoform B	8	7	9.91E+02	2.20E-08	E2RCX9*	1.822
Others	similar to CG5913-PA	6	5	6.97E+02	6.20E-04	E2R4W1*	0.580
Others	similar to heat shock 70kDa protein 8 isoform 2	40	5	4.68E+03	1.20E-05	E2R0T6*	0.399
Others	Structural maintenance of chromosomes protein; Structural maintenance of chromosomes protein 4	7	6	8.00E+02	1.80E-05	E2RCX4	0.448
Others	Transferrin receptor protein 1	9	8	9.97E+02	8.10E-03	F1PEN6*	0.530
Others	Trans-Golgi network integral membrane protein 2	3	3	5.00E+02	4.50E-05	E2RL53*	1.640
Others	Translationally-controlled tumor protein	6	5	7.97E+02	2.40E-10	E2RMA1	0.328
Others	Treacher Collins-Franceschetti syndrome 1	11	10	1.29E+03	9.00E-04	Q95KU4*	1.643
Others	Ubiquitin-associated protein 2	12	9	1.20E+03	1.10E-07	J9PB94	0.507
Others	ubiquitin-like modifier activating enzyme 1	23	15	2.65E+03	3.70E-04	E2RGH5	0.636
Others	Voltage-dependent anion-selective channel protein 1	13	11	1.60E+03	2.30E-07	F1PC72*	0.648

Others	WD repeat-containing protein 1	12	11	1.70E+03	2.00E-05	F1PR93	0.654
Proteasome	26S protease regulatory subunit 4	10	6	1.30E+03	1.60E-06	F1PQ40	0.631
Proteasome	proteasome (prosome, macropain) 26S subunit, ATPase, 4	8	6	1.28E+03	7.90E-03	E2RH48	0.628
Proteasome	proteasome (prosome, macropain) 26S subunit, non-ATPase, 2	17	16	2.30E+03	5.20E-10	E2RCP9	0.636
Proteasome	Proteasome activator complex subunit 3	7	5	8.98E+02	1.00E-05	E2RPY9	1.577
Proteasome	Proteasome-associated protein ECM29 homolog	8	5	7.88E+02	2.30E-03	F1PYA6	0.484
Protein transport	RAB3D, member RAS oncogene family	4	3	4.00E+02	4.70E-06	E2RFR4	0.488
Protein transport	syntaxin 7	3	3	2.99E+02	1.60E-07	E2R4Q2	0.462
Protein transpot	Nucleolar protein 58	9	6	1.10E+03	3.10E-04	E2RQV7	2.014
Protein transpot	Paralemmin-2	15	13	1.99E+03	4.20E-12	J9NTM8	3.801
Proteolysis	Cytosol aminopeptidase	18	17	2.40E+03	2.80E-07	E2RLY5	1.538
Proteolysis	Methylthioribulose-1-phosphate dehydratase	6	5	5.98E+02	2.60E-03	F1PT95	1.444
Proteolysis	secernin 2	3	3	4.00E+02	1.20E-06	E2QTH4	1.816
Redoxstatus	Catalase (Canis familiaris)	6	5	7.00E+02	1.60E-10	O97492	0.366
Redoxstatus	Glutathione S-transferase theta-1	5	4	5.87E+02	2.40E-07	J9NUJ7	0.641
Redoxstatus	Peroxiredoxin-2	7	5	6.99E+02	8.00E-05	F1PCG4	0.688
Redoxstatus	Protein disulfide-isomerase TMX3	8	5	7.97E+02	2.00E-06	F1P8Y7*	2.057
Redoxstatus	similar to Glutathione S-transferase P (GST 7-7) (Chain 7) (GST class-pi)	10	6	1.10E+03	3.10E-03	F1Q0J0*	2.103
Redoxstatus	Thioredoxin reductase 1, cytoplasmic	8	5	7.99E+02	6.30E-08	F1PBZ4	2.904
Signaling pathways	14-3-3 protein gamma	7	3	8.97E+02	5.70E-04	J9NRH5	1.471
Signaling pathways	Adapter molecule crk	6	6	6.00E+02	1.90E-08	E2QWD3	2.279
Signaling pathways	A-kinase anchor protein 12	40	34	4.97E+03	1.10E-06	F1PGL8	0.430
Signaling pathways	CAP, adenylate cyclase-associated protein 1 (yeast)	16	16	2.76E+03	3.30E-07	E2QZ50	0.476
Signaling pathways	Cell cycle and apoptosis regulator protein 2	12	11	1.20E+03	2.70E-05	E2RKJ1	1.467
Signaling pathways	cell division cycle 2, G1 to S and G2 to M	8	6	7.89E+02	4.70E-06	E2RGJ9	0.531

Signaling pathways	Cell growth-regulating nucleolar protein	5	5	5.98E+02	1.30E-06	E2RHX1	2.026
Signaling pathways	Condensin complex subunit 2	4	4	5.00E+02	7.90E-07	F1Q3I3	0.625
Signaling pathways	Cysteine-rich protein 2	4	4	5.00E+02	5.60E-04	F1PP40	0.697
Signaling pathways	Dihydropyrimidinase-related protein 2	19	16	2.20E+03	1.30E-10	F1P9U4	0.461
Signaling pathways	Dihydropyrimidinase-related protein 3	14	7	1.40E+03	3.40E-12	F1Q3Y2	0.194
Signaling pathways	DNA replication licensing factor MCM4	7	5	6.97E+02	4.30E-04	E2QSM6*	0.587
Signaling pathways	DnaJ homolog subfamily C member 2	5	5	4.99E+02	4.20E-08	E2RS54	0.541
Signaling pathways	Emerin	6	4	5.94E+02	5.70E-05	D0VWQ5	1.743
Signaling pathways	Glycylpeptide N-tetradecanoyltransferase 1	6	3	6.00E+02	1.60E-03	E2RPZ9	1.440
Signaling pathways	Microtubule-actin cross-linking factor 1 (Mus musculus)	19	14	1.90E+03	1.90E-04	F1Q1P4*	0.667
Signaling pathways	Microtubule-associated protein 4; Microtubule-associated protein	32	23	3.90E+03	1.30E-07	F1PCX8*	0.632
Signaling pathways	Microtubule-associated protein RP/EB family member 1	10	7	1.30E+03	1.10E-06	E2QYH4*	0.524
Signaling pathways	minichromosome maintenance complex component 3	10	9	1.20E+03	1.10E-03	E2RF73	0.663
Signaling pathways	Mitotic interactor and substrate of PLK1 (Pongo abelii)	12	11	1.39E+03	3.10E-07	J9NX80	0.681
Signaling pathways	non-SMC condensin I complex, subunit D2	12	8	1.20E+03	1.50E-02	E2RHZ0	0.624
Signaling pathways	Nucleosome assembly protein 1-like 1	7	6	9.00E+02	4.00E-02	F1PRE1	0.675
Signaling pathways	PDS5, regulator of cohesion maintenance, homolog A (S. cerevisiae)	10	9	9.89E+02	3.90E-05	E2R7R4	0.583
Signaling pathways	Peptidyl-tRNA hydrolase 2, mitochondrial	9	7	9.99E+02	3.60E-07	Q9GL24	3.172
Signaling pathways	platelet-activating factor acetylhydrolase, isoform Ib, subunit 1 (45kDa)	8	4	9.89E+02	1.60E-05	E2QY31*	1.947
Signaling pathways	Presequence protease, mitochondrial	10	7	1.19E+03	2.00E-06	F1PAM3	1.739
Signaling pathways	Prostaglandin E synthase 3	4	4	5.00E+02	2.80E-02	J9NT37	0.599

Signaling pathways	Protein LSM14 homolog A	4	4	5.00E+02	4.20E-04	F1PSJ7*	0.627
Signaling pathways	Protein S100-A10	5	3	7.91E+02	1.40E-02	J9NYC2	0.649
Signaling pathways	Ras GTPase-activating-like protein IQGAP1	36	33	4.39E+03	4.10E-10	F1PJ65	0.585
Signaling pathways	Ras-related protein Rab-10	6	4	5.96E+02	4.60E-05	F2Z4P9;*	0.591
Signaling pathways	Ras-related protein Rab-21	6	4	6.00E+02	8.00E-05	F1PGI1*	0.565
Signaling pathways	SEC14-like protein 2	8	7	8.89E+02	1.30E-04	E2RN29	1.493
Signaling pathways	Sister chromatid cohesion protein PDS5 homolog B	3	3	3.00E+02	5.50E-07	F1Q0Z0	2.170
Signaling pathways	solute carrier family 9 (sodium/hydrogen exchanger), member 3 regulator 1	15	13	1.68E+03	3.10E-06	E2RGF3	1.615
Sterol biosynthesis	Farnesyl pyrophosphate synthase	12	10	1.50E+03	7.60E-08	J9P2L3	1.613
Sterol biosynthesis	similar to Cytochrome P450 51A1 (CYPLI) (P450LI) (Sterol 14-alpha demethylase)	6	4	5.96E+02	5.70E-07	E2RB98	0.575
Sterol biosynthesis	squalene epoxidase	5	4	5.00E+02	5.30E-10	E2RHB9	0.204

*representative accession
