# Supplementary material for: Tracking changes in adaptation to suspension growth for MDCK cells: cell growth correlates with levels of metabolites, enzymes and proteins
Source: Appl Microbiol Biotechnol. 2021 Feb 13;105(5):1861–74. doi: 10.1007/s00253-021-11150-z (PMC7907048; doi:10.1007/s00253-021-11150-z)
Supplement: Supplementary file 2 — (PDF 60.1 kb) [file 253_2021_11150_MOESM2_ESM.pdf]

```
********** MODEL NAME
SCG-Model, MDCK SUS

********** MODEL NOTES
Segregated model to resemble suspension cell growth:

Original Model Development Reference (MDCK adherent):
The relation of growth phases, cell size changes and metabolism of adherent
cells
M. Rehberg, J. R. Ritter, Y. Genzel, D. Flockerzi, U. Reichl
Biotechnology & Bioengineering, 2012


Notes:
Units are given wherever possible at the right part.

********** MODEL STATES
d/dt(X1)          = + 2*r_trans*X<Nc> - r_trans*X1*f
d/dt(X2)          = + r_trans*X1*f - r_trans*X2
d/dt(X<idx,3:Nc>) = + r_trans*X<idx-1> - r_trans*X<idx>
d/dt(Glc)         = (- r_xGlc - r_mGlc) + k_evap*Glc/VM
d/dt(Gln)         = (- r_xGln - r_mGln - r_dGln) + k_evap*Gln/VM
d/dt(Glu)         = -r_Glu + k_evap*Glu/VM
d/dt(Lac)         = (+ r_xGlc + r_mGlc)*Y_LacGlc + k_evap*Lac/VM
d/dt(NH4)         = (+ r_xGln + r_mGln)*Y_NH4Gln + r_dGln  + r_Glu  +
k_evap*NH4/VM

X1(0)             = 3.9901e+05
X2(0)             = 0
X<idx,2:Nc>(0)    = 0
Glc(0)            = 31.0367
Gln(0)            = 1.6086
Glu(0)            = 3
Lac(0)            = 3.2500
NH4(0)            = 0
********** MODEL PARAMETERS
Nc                = 5            %-
s                 = 4.14699      %-
d_b               = 13.3269      %microm
d_c               = 1
Km                = 0.999884     %mM
mu_max            = 0.0137214    %1/h
VW                = 4e-3         %L
VC_max            = 12.9483      %microL
k_dGln            = 0            %1/h
k_evap            = 0            %L/h
m_Glc             = 0.212e-2     %mM/microL/h
m_Gln             = 5.29e-9      %mM/microL/h
Y_LacGlc          = 0.938749     %-
Y_NH4Gln          = 1.8
Y_xGlc            = 7.45167e-07 %mM/cells
Y_xGln            = 3.07336e-07 %mM/cells
k_GLT             = 1.92184e-14 %1/min
kbin2             = 1
```

```
********** MODEL VARIABLES
X_t                = X1+X2+X3+X4+X5                      %cells
di<idx,1:Nc>       = (d_b+(d_c-d_b)/(Nc-1)*<idx-1>)*X<idx>/X_t %microm
d                  = di1+di2+di3+di4+di5                       %microm
VC                 = 4/3*pi*(d/2/10^5)^3*10^6*X_t              %microL
mu                 = mu_max*Glc/(Km+Glc)                       %1/h
r_trans            = mu*(1/(2^(1/Nc)-1))                       %1/h
r_dGln             = Gln*k_dGln                                %mM/h
f                  = (1-exp(-s*(VC_max-VC)/VC))*kbin2          %-
VM                 = VW                                        %L
lambda             = 1                                         %-
r_xGlc             = mu*(X_t-X1+X1*f)*Y_xGlc*lambda            %mM/h
r_xGln             = mu*(X_t-X1+X1*f)*Y_xGln*lambda            %mM/h
r_mGlc             = m_Glc*VC*lambda                           %mM/h
r_mGln             = m_Gln*VC*lambda                           %mM/h
CSV                = VC*1e-6/X_t
Kmax_GLT           = k_GLT/CSV
r_Glu              = Kmax_GLT*Glu*(1-f)

r_GLC              =(r_xGlc+r_mGlc)*VM*1e6/VC
r_GLN              =(r_xGln+r_mGln)*VM*1e6/VC
r_GLUVC            = r_Glu*VM*1e6/VC
rx                 = mu_max
********** MODEL REACTIONS

********** MODEL FUNCTIONS

********** MODEL EVENTS
event1=ge(VC,VC_max),kbin2,0

********** MODEL MATLAB FUNCTIONS
```
